# Systematic Exploitation of Multiple Receptor Conformations for Virtual Ligand Screening

**DOI:** 10.1371/journal.pone.0018845

**Published:** 2011-05-17

**Authors:** Giovanni Bottegoni, Walter Rocchia, Manuel Rueda, Ruben Abagyan, Andrea Cavalli

**Affiliations:** 1 Department of Drug Discovery and Development (D3), Istituto Italiano di Tecnologia, Genova, Italy; 2 Skaggs School of Pharmacy and Pharmaceutical Sciences, University of California San Diego, La Jolla, California, United States of America; 3 Dipartimento di Scienze Farmaceutiche, Università di Bologna, Bologna, Italy; Consejo Superior de Investigaciones Cientificas, Spain

## Abstract

The role of virtual ligand screening in modern drug discovery is to mine large chemical collections and to prioritize for experimental testing a comparatively small and diverse set of compounds with expected activity against a target. Several studies have pointed out that the performance of virtual ligand screening can be improved by taking into account receptor flexibility. Here, we systematically assess how multiple crystallographic receptor conformations, a powerful way of discretely representing protein plasticity, can be exploited in screening protocols to separate binders from non-binders. Our analyses encompass 36 targets of pharmaceutical relevance and are based on actual molecules with reported activity against those targets. The results suggest that an ensemble receptor-based protocol displays a stronger discriminating power between active and inactive molecules as compared to its standard single rigid receptor counterpart. Moreover, such a protocol can be engineered not only to enrich a higher number of active compounds, but also to enhance their chemical diversity. Finally, some clear indications can be gathered on how to select a subset of receptor conformations that is most likely to provide the best performance in a real life scenario.

## Introduction

For over 20 years, High-Throughput Screening (HTS) has been one of the leading hit identification strategies in drug discovery [1]. Despite recent technological advances, HTS is still very expensive in terms of infrastructure, consumables, and personnel [2], being mainly carried out at the industrial level. Furthermore, HTS has a relatively high rate of false positives and false negatives, and is limited to comparatively small screening libraries. In this regard, Virtual Ligand Screening (VLS) represents a fast and cost-effective alternative, in which much larger libraries are screened by computational means [3], [4]. Compounds are assigned a predicted activity profile and are ranked accordingly. Experimental tests can be limited to the topmost ranking fraction of the compounds where, if the predictions are correct, the majority of active molecules will have been placed. VLS protocols can also be devised to improve “early recognition”, namely to increase the number of active compounds that are prioritized for testing [5], and to catch the broadest possible chemical diversity. Strategies to achieve these improvements may vary depending on the *in silico* approach to VLS. Usually, when a high quality crystallographic structure of the target (or its homologue) is available, structure-based strategies represent a suitable alternative to ligand- and pharmacophore-based methods [6]. Compound libraries are screened by iterating standard docking procedures against a target of interest, and the estimated binding score is used to prioritize putative hits. Processing massive libraries in a reasonable amount of time requires the introduction of several simplifications [7]–[9] and approximations [10], [11], which sometimes lead to low-accuracy predictions [12]–[14]. The use of a single receptor conformation is one of the major limitations that can hamper the quality of results [15]. This is particularly detrimental for early recognition since active compounds will act as true binders only in the presence of the right receptor conformation [16], [17].

Recently, many different implementations have been proposed to take into account protein flexibility in molecular docking and screening [18]. Multiple Receptor Conformations (MRC) is a straightforward and intuitive way to discretely mimic target plasticity [19]. In MRC docking, also known as ensemble docking, each putative ligand is docked separately at each receptor conformation, and the poses obtained in the independent runs are merged together. The predicted bound pose is assumed to be the one providing the overall best score. Several groups have extended the idea of MRC docking to VLS to increase, through receptor flexibility, the number of retrieved active molecules. The MRC paradigm can be applied to experimentally solved structures, computationally generated conformers, or both [20]. Remarkably, most of the studies only included multiple crystallographic structures. In these cases, protein plasticity could be directly inferred and no further validation was required. For instance, a seminal paper by Knegtel and coworkers reported attempts to increase the enrichment of known binders by using several crystallographic structures for both HIV protease and ras p21 [21]. For the HIV protease inhibitors, MRC docking systematically outperformed single conformer runs. In the case of ras p21, MRC docking performed better than the average, but it was outperformed several times by certain specific receptor conformations. Interestingly, the authors pointed out that, in a real (non-retrospective) screening campaign, there is no way to tell in advance which conformers are going to provide an optimal separation. Other studies have reported similar results when analyzing different systems and employing different docking engines [22]–[26]. In particular, Cavasotto and Abagyan have demonstrated how a limited receptor flexibility can more greatly affect the score determination (and thus the early recognition) than the reproduction of ligand-protein x-ray complexes [27]. Barril and Morley carried out a detailed analysis on the role of binding pocket flexibility in ligand docking [28]. They observed that an ensemble consisting of two conformations was enough to improve the enrichments in the topmost 1% fraction. The addition of further conformations did not significantly improve the performance and could even deteriorate it. Finally, Craig and coworkers tested the efficacy of MRC docking using BACE1 and cAbl with a challenging and purposely compiled benchmark [29]. Interestingly, their results were analyzed in terms of both enrichment and chemical diversity. In this context, we also carried out studies on MRC, focusing on the protocol's ability to reproduce ligands' x-ray poses, and taking into account induced fit effects. We first used a specific but highly challenging structural set [30], [31], and then a more comprehensive benchmark [32]. This latter set was further exploited to devise some practical rules for identifying optimal receptor conformation subsets [33].

Here, the beneficial role of x-ray MRC in VLS campaigns is explored systematically, carrying out retrospective screening studies against 36 well-known pharmacological targets. To improve accuracy, a set of drug-like ligands, compiled independently from the receptors, was carefully selected. The results are reported according to 5 robust figures of merit and evaluated by means of the following criteria: i) the separation power (binders from non-binders) as a function of receptor conformations; ii) the MRC-VLS performance compared to single conformation protocols (SRC-VLS) in terms of both number of active molecules and their chemical diversity; iii) contribution of each single receptor conformation to the MRC-VLS overall performance.

## Materials and Methods

### Benchmark Composition

The benchmark was obtained by selecting multiple high quality crystallographic structures for 36 pharmaceutically relevant targets from the Directory of Useful Decoys (DUD) [34], [35]. In the release adopted here, the original DUD set was filtered and annotated to avoid an artificial enrichment due to chemical redundancy. The crystal structures of targets were selected according to the criteria outlined to compile the previously reported experimental section of the flexible Pocketome and the 4D docking dataset [32], [36]. In the present study, the ability to provide a near-native pose for the cognate ligand was excluded from the filtering criteria. Four targets from DUD were excluded from the selection: HIV-1 Integrase (UniProt: P35963 – POL_HV1BR) and Peroxisome Proliferator-Activated Receptor γ Ligand Binding Domain (UniProt: P37231 – PPARG_HUMAN) due to the low number of ligands that were included in the adopted release of DUD, Human S-Adenosyl Homocysteine Hydrolase (UniProt: P23526 – SAHH_HUMAN) and β-type Platelet-derived Growth Factor Receptor (UniProt: P09619 – PGFRB_HUMAN) due to the lack of multiple high quality crystallographic structures available.

### Preparation of Receptor Structures

The correct atom types were assigned according to a modified version of ECEPP/3 force field [37]. Hydrogen atoms and missing heavy atoms were added. Zero occupancy side chains were optimized and assigned the lowest energy conformation. Tautomeric states of Histidines and the positions of Asparagine and Glutamine side chain amidic groups were optimized to improve the hydrogen bond pattern. Polar hydrogen atoms were also optimized. We deleted water molecules together with chains, heteroatoms, and prosthetic groups not involved in the binding site definition. The cognate ligands were deleted from the complexes only after hydrogen optimization.

### Preparation of Ligand Structures

3D atomic coordinates, tautomeric forms, stereochemistry, hydrogen atoms, and protonation states were assigned to ligands according to DUD. Each ligand was assigned MMFF force field atom types and charges [38].

### Binding Pocket Definition

The boundaries of the binding box were assumed to be known and directly derived from co-crystallized ligand coordinates. To achieve a common definition of the binding pocket, the receptor structures were superimposed using backbone atoms within 3.5 Å from the ligands. The iterative superimposition algorithm adopted here assigns different weights to different atomic subsets, gradually approaching the best solution for aligning the template and the other structures [39]. All residues with at least one side chain heavy atom in the range of 3.5 Å from any of the ligands belonging to the same ensemble were considered part of a common definition of the binding pocket. In this way, binding pocket could be defined consistently across the same structural ensemble, varying in conformation but not in composition.

### Single Conformer VLS

A standard screening run was carried out independently on each target conformer (single receptor conformation procedure or SRC). The docking engine used was the Biased Probability Monte Carlo (BPMC) stochastic optimizer as implemented in ICM (Molsoft LLC, La Jolla) [40]–[42]. The ligand binding site at the receptor was represented by pre-calculated 0.5 Å spacing potential grid maps, representing van der Waals potentials for hydrogens and heavy-atoms, electrostatics, hydrophobicity, and hydrogen bonding, respectively. The van der Waals interactions were described by the 6–12 Lennard-Jones potential. However, since the 6–12 standard implementation is extremely sensitive to even small deviations in atomic coordinates and can generate a large amount of noise in the intermolecular energy calculations, the default ICM docking procedure implements a smoother form of the Lennard-Jones potential, capping the repulsive contribution to 4 kcal/mol. A distance-dependent dielectric function was used (dielectric constant set equal to 4.0). Given the number of rotatable bonds in the ligand, the basic number of BPMC steps to be carried out was calculated by an adaptive algorithm [39]. The binding energy was assessed with the standard ICM empirical scoring function [40]–[44].

### Combining Results from Individual Runs

Several combinations of the results of the individual runs were probed to identify the most effective one in discerning actual binders from decoys. Results from independent runs were merged in one list and then re-ranked according to a descriptor inherited from individual runs. This post-processing step was carried out by means of ICM tables, data structures that allow storing, sorting, duplicates removal, and, in general, database-like handling of docking results. Re-ranking according to the best score (MRC-score) was a straightforward procedure: individual scores were merged into one list, which was then sorted in ascending order. In the unlikely case that two molecules achieved exactly the same score, the molecule displaying lower molecular weight achieved a better rank. As an alternative, we used the combination obtained by re-ranking all ligands according to the best rank that each compound obtained across individual runs (MRC-rank). This procedure is not univocal (there are possibly *n* molecules ranking first if *n* runs are carried out) and therefore the list was processed again so that molecules with the same best rank were then sorted according to their score.

### Figures of Merit

The literature contained several metrics for evaluating the effectiveness of a docking run in discriminating actual binders from decoys, some of them addressing the issue of the early recognition [5]. For evaluating the performance of different combinations of protein conformers, we considered: the Area Under the Accumulation Curve (AUAC), the area under the Receiver Operating Characteristic curve (ROC), the Enrichment Factor (EF) [45] at different thresholds, the Robust Initial Enhancement (RIE) [46], and the Boltzmann-Enhanced Discrimination of ROC metric (BEDROC) [5]. RIE as well as BEDROC needed the assignment of a parameter, termed alpha, for which we chose a value of 20, as suggested by the literature [5], [46].

All 5 metrics rely on the so-called *accumulation curve*, F_a_(k), where the subscript “a” stands for active molecule, which represents the count, possibly normalized, of how many true binders obtained a rank better or equal to a given one in a docking run; F_a_(k) contains all the information needed to assess the performance of a run, and a combination of multiple runs as well.

The AUAC is the area under the chart of F_a_(k) and, in its discretized version, takes the following expression: 
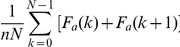
, where n is the number of actual binders and N the total number of screened molecules. It ranges from 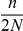 to 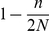, where the higher the value, the better the performance.

The ROC curve is a widely used way of representing the same information; it plots the number of actual binders with respect to the inactive molecules found in a docking run. It takes the following expression: 
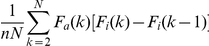
, where the subscript “i” stands for inactive molecule. It can be shown that the area under the ROC curve is a linear transformation of the AUAC, and more convenient since it ranges from 0 to 1. It follows that they share the same information content and can be used interchangeably.

The EFχ is the measure of how many more binders are found within a predefined “early recognition” fraction χ of the ordered list relative to a random distribution. Its expression can be recast in this concise formula: 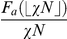, where the 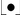 lower brackets symbol stands for the greatest integer lower or equal to the argument. It ranges from a minimum value of 0 to a maximum of 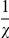 if 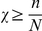 or 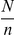 otherwise.

RIE is an early recognition metric that uses a decreasing exponential weight as a function of rank. This exponential smoothing should make RIE a more robust metric with respect to EF when a small number of actives are considered. The counterpart of the 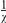 quantity for EF is the α parameter of the exponential smoothing. The discretized form of RIE is: 
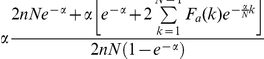
 and its range is 
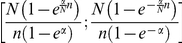
.

Finally, the BEDROC metric was defined as a standardization of the RIE so that it ranges from 0 to 1, and in fact it can be expressed as: 
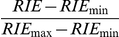
. It appears clear from its definition that it contains the same information as RIE, so they can be used interchangeably.

### Software and Hardware

The receptor and ligand preparations, the virtual ligand screening simulations, and the energy evaluations were carried out with ICM 3.7 (Molsoft L.L.C., La Jolla, CA). The statistical analysis and figures of merit were calculated with purposely developed in-house scripts in MATLAB v.7-R14 (MathWorks, Natick, MA).

The hardware facilities used in the present study were a dual Quad-Core AMD Opteron™ “Barcelona” workstation and a 42 Quad/Esa-Core 64-bit AMD Opteron™ “Istanbul/Shanghai” computer cluster.

## Results and Discussion

### I. The Dataset

The MRC-VLS simulations were carried out using two independent datasets: DUD and the experimental Flexible Pocketome. Thirty-six pharmaceutically relevant targets were retrieved. For each of them, a series of conformations (Pocketome) together with a set of known binders and *bona fide* non-binders (DUD) were available. In particular, 2 to 30 conformers were collected for each target (overall 457 high quality crystal structures). The median intra family RMSD of the side chain's heavy atoms of the binding site was 1.6 Å while the median intra family RMSD of the backbone was 1.0 Å. Ligand sets encompassed from 8 to 365 known binders and from 155 to 15,560 decoys. Due to the filtering procedures introduced in the current version of DUD [34], the average ratio between binders and decoys was not fixed but varied slightly, with an average value of 0.023 (1∶43), slightly lower than the original 0.028 (1∶36). The set includes 6 nuclear receptors and 29 enzymes comprising proteases, hydrolases, kinases, etc. Details about MRC-VLS benchmark are reported in Table 1, while a complete list of the PDB structures included in the MRC-VLS test set is reported in the Supporting Information (Table S1).

**Table 1:** The complete MRC-VLS test set.

TARGET	Conformers	Binders	Decoys	Total Ligands	RatioBinders/Non Binders	Binders Chemotypes
ACE_HUMAN	7	46	996	1042	0.046	18
ACES_TORCA	21	99	3859	3958	0.025	18
ADA_BOVIN	13	23	927	950	0.024	8
ALDR_HUMAN	15	46	1796	1842	0.025	14
AMPC_COLI	16	21	786	807	0.026	6
ANDR_HUMAN	29	68	2848	2916	0.023	10
CDK2_HUMAN	30	47	2070	2117	0.022	32
COMT_RAT	3	11	468	479	0.023	2
DHFR_HUMAN	6	190	8350	8540	0.023	14
EGFR_HUMAN	6	365	15560	15925	0.023	40
ESR1_AG_HUMAN	4	63	2568	2631	0.024	10
ESR1_ANT_HUMAN	13	18	1058	1076	0.017	8
F10A_HUMAN	20	64	2092	2156	0.030	19
FGFR1_HUMAN	4	71	3462	3533	0.020	12
GCR_HUMAN	4	32	2585	2617	0.012	9
HMDH_HUMAN	9	25	1423	1448	0.017	4
HS9A_HUMAN	20	23	975	998	0.023	4
INHA_MYCTU	14	57	2707	2764	0.021	23
KITH_HHV11	19	22	891	913	0.025	7
MCR_HUMAN	11	13	636	649	0.020	2
MK14_MOUSE	19	137	6779	6916	0.020	20
NRAM_INBBE	11	49	1713	1762	0.028	7
PARP1_CHICK	6	31	1350	1381	0.023	7
PDE5A_HUMAN	11	26	1698	1724	0.015	22
PGH1_SHEEP	2	23	910	933	0.025	11
PGH2_MOUSE	2	212	7632	7844	0.027	44
PNPH_BOVIN	19	25	1036	1061	0.024	4
POL_HV1RT	18	34	1494	1528	0.022	17
PRGR_HUMAN	6	22	920	942	0.024	4
PUR3_COLI	3	8	155	163	0.051	5
PYGM_RABIT	20	52	2135	2187	0.024	10
RXRA_HUMAN	15	18	575	593	0.031	3
SRC_HUMAN	14	98	5679	5777	0.017	21
THRB_HUMAN	20	23	1148	1171	0.020	14
TRY1_BOVIN	19	9	718	727	0.012	7
VGFR2_HUMAN	8	48	2712	2760	0.017	31

Here, we wanted to explore MRC-VLS capabilities against a set of ligands compiled independently from the receptors. The choice to combine already reported test sets for receptors and ligands rather than compiling a new one from scratch reflected an endorsement of the growing request in the field of VLS to adopt accepted and shared standards [47]. To the best of our knowledge, this is the most comprehensive test set where experimental protein structures and known ligands are used together to explore the role of MRC in a VLS study retrospectively. Finally, it should be mentioned that the non-native protein conformer dataset, compiled by Verdonk and coworkers [48] and extending the approach that led to the Astex Diverse Set [49], could represent a valid and appropriate alternative source of receptor variants for MRC-VLS validation.

### II. Binder Distribution in SRC-VLS runs

**Figure 1:**
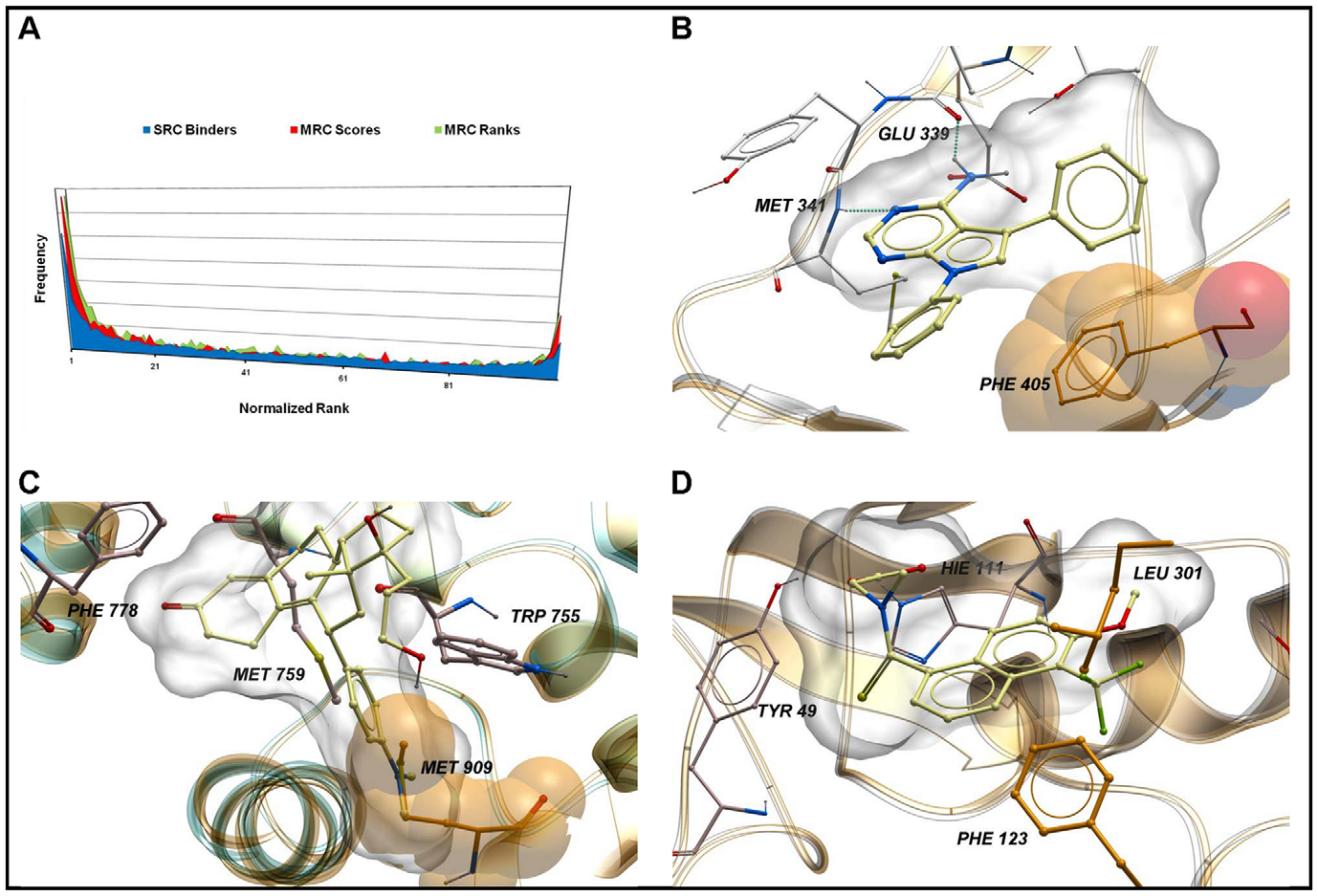
The role of induced fit in MRC VLS. A) Frequency distributions of SRC binders (blue area), MRC score binders (red area), and MRC rank binders (green area) with respect to the relative rank they obtained in individual VLS runs. Two peaks emerge in the binders' distribution: the highest one, on the left, corresponds to the expected behavior where binders rank among the best positions, while the peak on the rightmost part of the distribution corresponds to the opposite phenomenon. B) Inhibitor **1** at the binding site of SRC kinase. The structure that can accommodate the ligand is reported in transparent green ribbons and the structure incompatible with the native binding mode in grey. The boundaries of the binding site are highlighted by a semi-transparent white mesh. Inhibitor **1** and the interacting residues are reported explicitly in ball and stick representation and labeled. Carbon atoms of inhibitor **1** are light yellow and carbon atoms of the binding site residues are light grey. The clashing residue Phe405 from the incompatible structure is reported explicitly in ball and stick representation with orange carbon atoms. The van der Waals volume of the clashing Phe405 is highlighted by an orange mesh. Intermolecular hydrogen bonds are reported with dotted lines. C) Modulator **2** at the binding site of Progesterone receptor. The structure that can accommodate the ligand is reported in transparent green ribbons and the structure incompatible with the native binding mode in grey. The boundaries of the binding site are highlighted by a semi-transparent white mesh. Modulator **2** and the binding site residues are reported explicitly in ball and stick representation and labeled. Carbon atoms of modulator **2** are light yellow and carbon atoms of the binding site residues are light grey. The clashing residue Met909 from the incompatible structure is reported explicitly in ball and stick representation with orange carbon atoms. The van der Waals volume of the clashing Met909 is highlighted by an orange mesh. D) Inhibitor **3** at the binding site of aldose reductase. The structure that can accommodate the ligand is reported in transparent green ribbons and the structure incompatible with the native binding mode in grey. The boundaries of the binding site are highlighted by a semi-transparent white mesh. Inhibitor **3** and the binding site residues are reported explicitly in ball and stick representation. Carbon atoms of inhibitor **3** are light yellow and carbon atoms of the binding site residues are grey. Clashing residues Phe146 and Leu300 from the incompatible structure are reported explicitly in ball and stick representation with orange carbon atoms. The van der Waals volumes of the clashing Phe123 and Leu301 are highlighted by orange meshes. Figure 1B, 1C, and 1D were rendered with ICM3.7.

First, we focused on the assessment of SRC runs by plotting the distribution of known binders against their relative rank (see Figure 1A). The distribution displayed two maxima. The plot of an ideal situation would present all binders located in the first positions. However, in the rightmost part of Figure 1A, another peak was observed. This showed that, in several cases, SRC-VLS was not only unable to rank known binders in the top scoring fraction but that these molecules ended up ranking lower than an *average* decoy. This behavior was mainly due to the fact that some receptor conformations could lodge certain ligands remarkably well but could dump those which required a different binding site rearrangement. For example, type I protein kinase inhibitor **1** (5,7 diphenylpyrrolo[2,3-d]pyrimidine, Figure 1B) ranked 6^th^ when screened using a conformation of the proto-oncogene tyrosine protein kinase SRC (SRC_HUMAN) co-crystallized in complex with a ligand very similar to **1** (PDBid: 1YOL, see also Figure S1A in the Supporting Information). Conversely, **1** ranked only 5,118^th^ when docked at the inactive, DFG-out conformation of the same protein when it is complexed with Imatinib (PDBid: 2OIQ). This first example clearly shows the fundamental role of taking into account several receptor conformations for relatively flexible protein families (such as kinases). In a further case study, a selective modulator (**2**) of the progesterone receptor (PRG_HUMAN) was ranked 2^nd^ when docked using the conformation solved in complex with Asoprisnil (PDBid: 2OVH). The exceptionally high score could be achieved since the N,N-dimethylanilino substituent of **2** could almost perfectly fill an accessory pocket created by the conformational rearrangement of Met909 side chain. Remarkably, in this case too, **2** and the ligand co-crystallized in 2OVH were structurally very similar (see also Figure S1B in the Supporting Information). When using a conformer lacking the Met909-based pocket (PDBid: 1ZUC), **2** could only be placed in position 920. Aldose reductase (ALDR_HUMAN) provided another example of how the binding site plasticity could affect VLS results. **3** (Tolrestat) ranked 1^st^ when docked at the binding site of its cognate receptor (PDBid: 1FZB). Due to a different rearrangement of the Leu300 and Phe122 side chains (Figure 1D), **3** was ranked 915^th^ in a VLS run carried out with a receptor conformation obtained from the crystal structure of the enzyme in complex with a different inhibitor (PDBid: 1T40, see also Figure S1C in the Supporting Information). As expected, the analysis of the results confirmed that true binders were ranked at the first positions, when a suitable receptor conformation was used. In these cases, SRC-VLS performed well in terms of early recognition. Conversely, SRC-VLS could not identify true binders when non-cognate receptor conformations (or similar) were used. In these cases, the overall performance of true binders was even worse than that of smaller decoys that could establish non-specific interactions. This is in good agreement with previous reports on the same topic [50], [51]. Overall, SRC-VLS outperformed the “random picking” baseline (as expected); however it was unable to guarantee a systematic ranking of true binders among the first hits. True binders employed in this study were selected independently from the receptor structures and annotated according to their reported experimental activity. Unfortunately, a high experimental affinity does not automatically translate into favorable binding scores in a docking or VLS experiment (and *vice versa*). Moreover, overlooking receptor flexibility is not the only reason that can lead to inaccurate predictions. Even in presence of a perfectly adapted receptor structure, docking simulations can fail mainly because of well known limitations and approximations introduced in sampling and scoring, extensively reported and discussed elsewhere [7]–[15]. Even in cross-docking, unexpected (if not counterintuitive) results can be produced. For instance, a ligand cannot be re-docked into its cognate receptor but can be accurately lodged in another structure of the same target co-crystallized with a different ligand [28]. Finally, it is worth to stress that decoys employed here are *bona fide* non-binders, since their lack of activity has not been proven experimentally. It would not be unheard of that a decoy scored consistently well because it is actually a binder [52].

**Figure 2:**
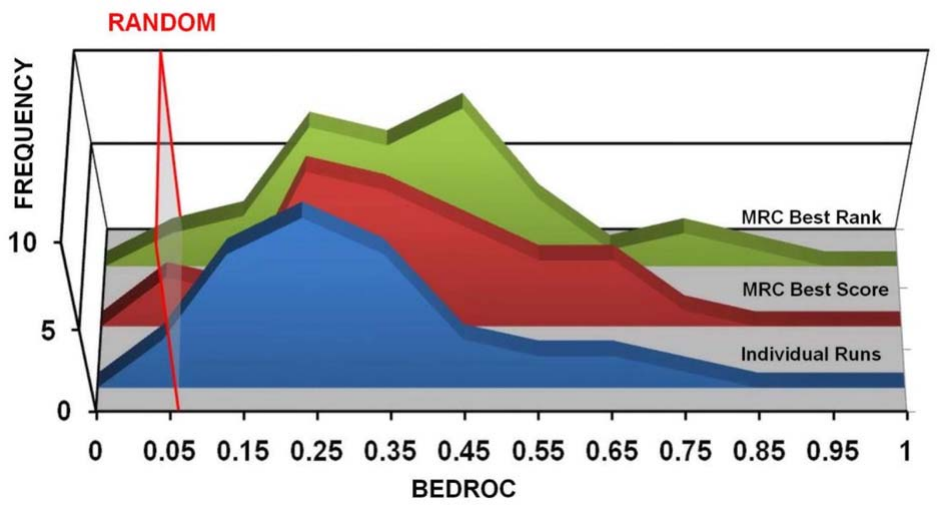
Frequency distributions describing the performance of different protocols, as assessed by the BEDROC metric, with α = 20. The red-bordered transparent grey rectangle represents the threshold of randomness. The blue area represents the frequency distribution of the results for the individual runs (average), the red area represents the frequency distribution of the results for the MRC-score, and the green area represents the frequency distribution of the results for the MRC-rank.

The SRC-VLS performance depended on the specific target and, to a lesser extent, on the figure of merit that was considered [23], [53]. For instance, using BEDROC figure of merit (blue plot in Figure 2), individual docking runs performed on average four times better than random picking.

### III. Multiple Receptor Conformations Results

The first outcome of this study was a comparison between MRC-VLS and SRC-VLS. For each target, all binders and decoys collected in DUD were screened against all the conformers of the receptor in the set. The MRC results were generated, as described in the Methods section, according to the MRC-score and MRC-rank combinations, and assessed using different figures of merit. In particular, we selected AUAC, EF, and BEDROC to reduce redundancy. For each of them, a frequency distribution of the results obtained by MRC protocols was compiled and the percentile in which these results fell was reported. For example, six crystallographic structures are available for the human progesterone receptor (PRGR_HUMAN). SRC-VLS runs carried out with each of them provided the following results in terms of AUAC: 0.72, 0.70, 0.65, 0.62, 0.61, and 0.61. The AUAC corresponding to the MRC-score protocol was 0.71, falling in the 85^th^ percentile and outperforming 5 SRC-VLS runs (out of 6). The AUAC corresponding to the MRC-rank protocol was 0.84, outperforming all the SRC-VLS runs and thus falling in the 100^th^ percentile. We would like to stress here that, for standard VLS runs (where experimental information about possible binders and decoys are missing), the conformation selection is a major issue because it can greatly affect the VLS results, and also because it is very hard to establish “a priori” which conformer is able to provide the best results [33]. Besides MRC-score and MRC-rank protocols, already described in the Methods section, we explored several different combination schemes, as briefly reported: i) using the second and third best ranks obtained by a MRC docking campaign to reorder ligands having the same best rank; ii) treating scores and ranks as putative energy estimators, using them in a Boltzmann combination; iii) a second Boltzmann combination that included molecular weights as a further ranking criterion; iv) testing the overall combination of the ranks provided by all of the other mentioned figures. None of these methods outperformed MRC-score or MRC-rank.

#### III.A. AUAC

**Figure 3:**
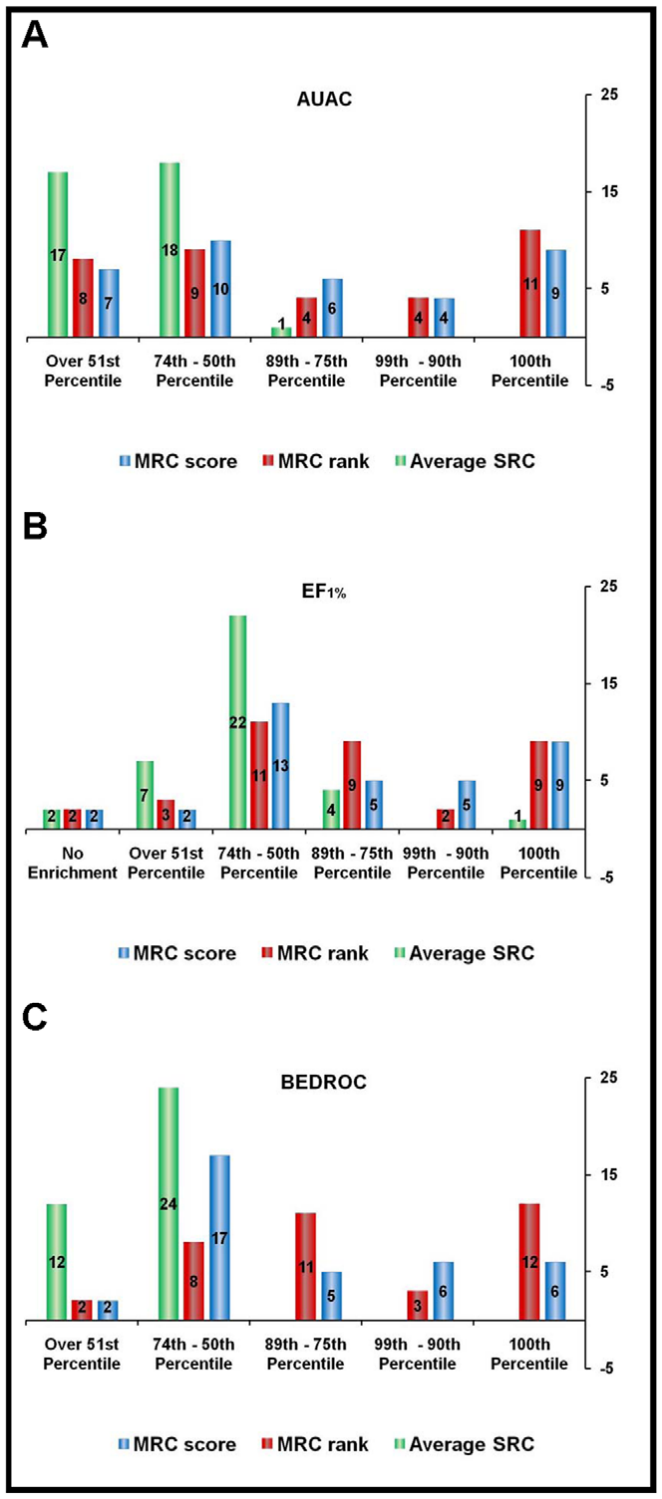
Distribution of the results of MRC-VLS runs according to different figures of merit. Blue histograms represent MRC-score results, red histograms represent MRC-rank results. The average SRC performance is reported (green histograms) as a term of comparison. A) Histograms representing the distribution of the results according to AUAC. B) Histograms representing the distribution of the results according to EF_1%_. C) Histograms representing the distribution of the results according to BEDROC.

Table 2 and Figure 3A show the performance of MRC-VLS protocol according to AUAC. In 9 out of 36 targets, MRC-score performed equally well or better than any single conformer (100^th^ percentile). It was placed between the 99^th^ and the 90^th^ percentile 4 times (i.e. for 4 targets), between the 90^th^ and the 75^th^ percentile 6 times, and between the 75^th^ and the 50^th^ percentile 10 times. For 5 targets, the MRC-score was placed below the 50^th^ percentile. On average, the MRC-score AUAC fell in the 70^th^ percentile or better, suggesting that multiple receptor conformations enhanced the ability of this protocol to separate binders from non-binders with respect to SRC-VLS. We then analyzed in detail the runs that were below the 50^th^ percentile. In particular, two contrary scenarios were observed: i) for 3 targets (PUR_ECOLI, PDE5A_HUMAN, and MCR_HUMAN), an exceptionally high performance of SRC-VLS with all conformers was detected, and these results could not be further improved by our MRC-VLS approach; ii) in contrast, for 2 targets (ADA_BOVIN and HMDH_HUMAN), we observed very poor performances (i.e. located below or barely above the threshold of randomness), which could not be improved even using the MRC-VLS protocol. ADA_BOVIN (i.e. adenosine deaminase) is a metalloenzyme involved in purine metabolism. It bears a very peculiar network of interactions at the binding site, which revolves around the coordination complex formed by a zinc ion, three hystidine residues, and the inhibitor. Furthermore, the binding pocket is quite large while the known ligands are relatively small. For these reasons, ADA_BOVIN is widely recognized to be a very complicated target for computational studies. As a matter of fact, in the original DUD study by Huang and colleagues [35], ADA_BOVIN was already reported as a target that did not provide any significant separation between binders and non-binders via a fully automated protocol. HMDH_HUMAN (i.e. HMG-CoA reductase) is an interesting example of how noise generated in individual runs can accumulate to compromise the global performance of MRC-VLS. In particular, for several receptor conformers, decoys systematically outranked true binders and were assigned very high scores which, in turn, inflated their final position in our MRC-VLS. Even though it is beyond the purpose of the present study, it should be pointed out that, exploiting the knowledge of an expert on the target biology, a customized tuning of the binding pocket composition and of the docking protocol parameters could significantly improve both the SRC and MRC VLS performances [54].

**Table 2:** SRC and MRC statistics in terms of AUAC.

TARGET	Number of Conformers	Min SRCAUAC	Max SRCAUAC	Mean SRCAUAC	MRC-scoreAUAC	MRC-rankAUAC	IdealAUAC
ACE_HUMAN	7	0.52	0.59	0.55	0.66	0.66	0.99
ACES_TORCA	21	0.43	0.73	0.55	0.67	0.63	0.99
ADA_BOVIN	13	0.20	0.39	0.28	0.15	0.17	0.99
ALDR_HUMAN	15	0.47	0.72	0.57	0.79	0.80	0.99
AMPC_COLI	16	0.28	0.59	0.45	0.47	0.44	0.98
ANDR_HUMAN	29	0.34	0.74	0.61	0.74	0.75	0.99
CDK2_HUMAN	30	0.52	0.81	0.65	0.82	0.81	0.99
COMT_RAT	3	0.51	0.58	0.54	0.54	0.55	0.99
DHFR_HUMAN	6	0.74	0.88	0.79	0.90	0.92	0.99
EGFR_HUMAN	6	0.41	0.65	0.51	0.54	0.65	0.99
ESR1_AG_HUMAN	4	0.71	0.82	0.76	0.82	0.82	0.99
ESR1_ANT_HUMAN	13	0.35	0.53	0.44	0.43	0.43	0.99
F10A_HUMAN	20	0.48	0.86	0.66	0.81	0.80	0.98
FGFR1_HUMAN	4	0.36	0.58	0.43	0.55	0.58	0.99
GCR_HUMAN	4	0.25	0.62	0.42	0.45	0.54	0.99
HMDH_HUMAN	9	0.60	0.77	0.65	0.58	0.61	0.99
HS9A_HUMAN	20	0.28	0.62	0.44	0.41	0.49	0.99
INHA_MYCTU	14	0.35	0.66	0.52	0.63	0.58	0.99
KITH_HHV11	19	0.40	0.75	0.6	0.74	0.72	0.99
MCR_HUMAN	11	0.80	0.87	0.85	0.82	0.84	0.99
MK14_MOUSE	19	0.28	0.70	0.47	0.61	0.62	0.99
NRAM_INBBE	11	0.79	0.90	0.85	0.88	0.86	0.99
PARP1_CHICK	6	0.67	0.79	0.73	0.83	0.83	0.99
PDE5A_HUMAN	11	0.65	0.84	0.77	0.77	0.77	0.99
PGH1_SHEEP	2	0.70	0.70	0.70	0.73	0.75	0.99
PGH2_MOUSE	2	0.58	0.75	0.67	0.71	0.69	0.99
PNPH_BOVIN	19	0.46	0.79	0.60	0.77	0.80	0.99
POL_HV1RT	17	0.46	0.68	0.61	0.72	0.76	0.99
PRGR_HUMAN	6	0.61	0.72	0.65	0.71	0.84	0.99
PUR3_COLI	3	0.80	0.88	0.85	0.85	0.94	0.99
PYGM_RABIT	20	0.21	0.44	0.32	0.22	0.26	0.99
RXRA_HUMAN	15	0.50	0.93	0.76	0.88	0.85	0.98
SRC_HUMAN	14	0.38	0.63	0.52	0.61	0.60	0.99
THRB_HUMAN	20	0.28	0.63	0.47	0.50	0.50	0.99
TRY1_BOVIN	19	0.33	0.89	0.69	0.76	0.79	0.99
VGFR2_HUMAN	8	0.45	0.62	0.55	0.70	0.73	0.99

In MRC-rank (Table 2), the results turned out to be slightly better than in MRC-score. A few significant differences were observed for the following targets: i) EGFR_HUMAN and PRGR_HUMAN AUAC's assessments were improved with respect to MRC-score; ii) the AUAC for GCR_HUMAN and HS9A_HUMAN improved with respect to the median target results in MRC-score; iii) PUR3_COLI could be moved from below the 50^th^ percentile in MRC-score (AUAC of 0.85) to the 100^th^ percentile in MRC-rank (AUAC of 0.94). The one-tailed t-student's test for paired samples assessed that both MRC-score and rank outperformed the average individual docking run with high significance, p<10^−5^.

#### III.B. EF

When the figure of merit considered was the EF_1%_ (Table 3 and Figure 3B), MRC-score was in the 100^th^ percentile for 9 targets. MRC-score results were placed between the 90^th^ and the 99^th^ percentile 5 times, between the 75^th^ and the 90^th^ percentile 5 times, and between the 50^th^ and the 75^th^ percentile 13 times. In only two examples, namely NRAM_INBEE and DHFR_HUMAN, was the combined EF_1%_ from MRC-score placed below the 50^th^ percentile. MRC-rank and MRC-score provided very similar results, with MRC-score performing better than MRC-rank when the instances that fell within the 90^th^ percentile were considered (14 for MRC-score and 11 for MRC-rank). It should be pointed out that, for ADA_BOVIN and COMT_RAT, both individual runs and MRC protocols systematically failed to provide any enrichment at 1%. For these two targets, MRC results were not included among those placed in the 100^th^ percentile since the fact that MRC performed equally well with respect to the best SRC did not appear particularly relevant.

**Table 3:** SRC and MRC statistics in terms of EF_1%_.

TARGET	Number of Conformers	Min SRC EF_1%_	Max SRC EF_1%_	Mean SRC EF_1%_	MRC-score EF_1%_	MRC-rank EF_1%_	Ideal EF_1%_
ACE_HUMAN	7	6.5	21.7	15.2	23.9	23.9	39.1
ACES_TORCA	21	0	16.1	2.2	4.0	5.0	39.0
ADA_BOVIN	13	0	0	0	0	0	39.1
ALDR_HUMAN	15	0	30.7	17.9	34.6	30.8	38.4
AMPC_COLI	16	0	4.7	0.6	0	4.7	38.0
ANDR_HUMAN	29	2.9	23.5	14.7	25.0	22.0	42.6
CDK2_HUMAN	30	2.1	21.2	10.3	10.6	12.7	44.6
COMT_RAT	3	0	0	0	0	0	36.3
DHFR_HUMAN	6	13.7	33.6	20.3	14.2	20.5	44.7
EGFR_HUMAN	6	3	16.1	9.3	9.9	14.0	43.5
ESR1_AG_HUMAN	4	14.3	19.0	16.2	17.4	12.7	41.2
ESR1_ANT_HUMAN	13	0	22.2	12.4	16.6	11.1	55.5
F10A_HUMAN	20	0	25.0	7.9	15.6	9.3	32.8
FGFR1_HUMAN	4	2.8	7.0	4.5	2.8	9.8	49.2
GCR_HUMAN	4	0	25.0	7.3	25.0	25.0	81.2
HMDH_HUMAN	9	4	36.0	17.7	20.0	24.0	56.0
HS9A_HUMAN	20	0	21.7	6.7	0	17.4	39.1
INHA_MYCTU	14	0	8.7	2.0	5.2	1.7	47.3
KITH_HHV11	19	0	9.1	2.6	9.1	4.5	40.1
MCR_HUMAN	11	30.7	46.1	39.8	38.4	30.7	46.1
MK14_MOUSE	19	0	13.1	3.15	11.7	7.3	50.3
NRAM_INBBE	11	4.1	28.6	16.9	16.3	10.2	34.7
PARP1_CHICK	6	3.22	16.1	8.0	9.7	6.4	41.9
PDE5A_HUMAN	11	3.8	23.0	11.1	15.3	11.5	65.4
PGH1_SHEEP	2	13	17.4	15.2	17.4	13.0	39.1
PGH2_MOUSE	2	0.9	3.8	2.3	2.3	2.3	36.8
PNPH_BOVIN	19	0	16.0	5.0	4.0	16	40.0
POL_HV1RT	18	2.9	20.6	10.5	11.7	14.7	44.1
PRGR_HUMAN	6	13.6	27.2	22.7	27.2	27.2	40.9
PUR3_COLI	3	12.5	12.5	12.5	12.5	12.5	12.5
PYGM_RABIT	20	0	3.8	0.9	1.9	1.9	40.3
RXRA_HUMAN	15	0	22.2	5.9	11.1	5.5	27.7
SRC_HUMAN	14	1.0	15.3	8.4	11.2	10.2	58.1
THRB_HUMAN	20	0	13.0	5.0	4.3	4.3	47.82
TRY1_BOVIN	19	0	11.1	2.9	0	11.1	77.7
VGFR2_HUMAN	8	4.1	18.7	8.1	18.7	12.5	56.2

EF_1%_ is a stringent figure of merit and yet, in all but a very few examples, MRC-VLS provided an early recognition that was better than or equal to most SRC runs. The one-tailed t-student's test for paired samples for EF_1%_ assessed that MRC-score and MRC-rank protocols outperformed the average individual docking run with a significance of p<0.0025 and p<0.001, respectively. With an EF_10%_ the improved performance of MRC-VLS was indeed evident: MRC-score and MRC-rank were above the 90^th^ percentile for 16 and 15 targets, respectively. For 27 targets, both MRC protocols were above the 75^th^ percentile. The complete results for EF_10%_ are reported in the Supporting Information (Table S2).

Comparing AUAC – a figure of merit for the overall performance – and EF – a figure of merit for the early recognition – we could confirm the general improvement in separating binders from non-binders, and we could show that this improvement was particularly marked in the topmost ranking fraction. This is particularly relevant for VLS protocols, where early recognition of true binders is a major achievement of this computational drug discovery approach.

#### III.C. BEDROC

MRC-VLS combines an increased ability to separate binders from non-binders with an improved propensity toward early recognition. This ability can be concisely described by adopting BEDROC as a figure of merit (see Methods). According to the frequency distribution of the results (Table 4 and Figure 3C), MRC-score outperformed or performed as well as the best of the single rigid conformers 6 times, was between the 99^th^ and the 90^th^ percentile 6 times, and between the 90^th^ and 75^th^ percentile 6 times. In 16 targets, MRC-score produced results that were between the 75^th^ and the 50^th^ percentile. PUR3_COLI and MCR_HUMAN were the only targets whose MRC-score was below the 50^th^ percentile. MRC-rank was in the 100^th^ percentile 12 times. It was between the 99^th^ and the 90^th^ percentile 3 times, between the 90^th^ and 75^th^ percentile 11 times, and between the 50^th^ and the 75^th^ percentile 8 times. In two cases, namely PDE5A_HUMAN and NRAM_INBBE, the MRC-rank was below the 50^th^ percentile despite being very close to the average of SRC-VLS performance.

**Table 4:** SRC and MRC statistics in terms of BEDROC.

TARGET	Number of Conformers	Min SRC BEDROC	Max SRC BEDROC	Mean SRC BEDROC	MRC-score BEDROC	MRC-rank BEDROC	Ideal BEDROC
ACE_HUMAN	7	0.23	0.37	0.31	0.45	0.44	1
ACES_TORCA	21	0.01	0.37	0.10	0.25	0.17	1
ADA_BOVIN	13	0	0.05	0.01	0.01	0.01	1
ALDR_HUMAN	15	0.04	0.47	0.3	0.66	0.66	1
AMPC_COLI	16	0.01	0.13	0.05	0.06	0.07	1
ANDR_HUMAN	29	0.12	0.45	0.32	0.45	0.45	1
CDK2_HUMAN	30	0.10	0.51	0.27	0.40	0.41	1
COMT_RAT	3	0.01	0.08	0.04	0.07	0.06	1
DHFR_HUMAN	6	0.38	0.71	0.48	0.55	0.64	1
EGFR_HUMAN	6	0.09	0.4	0.23	0.21	0.36	1
ESR1_AG_HUMAN	4	0.40	0.51	0.47	0.54	0.54	1
ESR1_ANT_HUMAN	13	0.01	0.33	0.22	0.30	0.28	1
F10A_HUMAN	20	0.05	0.59	0.26	0.53	0.38	1
FGFR1_HUMAN	4	0.08	0.25	0.14	0.17	0.22	1
GCR_HUMAN	4	0	0.30	0.11	0.27	0.28	1
HMDH_HUMAN	9	0.21	0.46	0.28	0.42	0.42	1
HS9A_HUMAN	20	0	0.37	0.11	0.05	0.23	1
INHA_MYCTU	14	0.01	0.20	0.09	0.19	0.12	1
KITH_HHV11	19	0.01	0.23	0.14	0.22	0.22	1
MCR_HUMAN	11	0.54	0.76	0.67	0.62	0.68	1
MK14_MOUSE	19	0.01	0.31	0.09	0.22	0.18	1
NRAM_INBBE	11	0.37	0.72	0.56	0.61	0.46	1
PARP1_CHICK	6	0.36	0.47	0.42	0.44	0.44	1
PDE5A_HUMAN	11	0.17	0.42	0.31	0.37	0.30	1
PGH1_SHEEP	2	0.25	0.31	0.28	0.30	0.33	1
PGH2_MOUSE	2	0.06	0.28	0.17	0.22	0.17	1
PNPH_BOVIN	19	0.03	0.49	0.2	0.31	0.39	1
POL_HV1RT	18	0.09	0.38	0.24	0.36	0.43	1
PRGR_HUMAN	6	0.24	0.49	0.39	0.53	0.54	1
PUR3_COLI	3	0.50	0.61	0.57	0.60	0.77	1
PYGM_RABIT	20	0	0.07	0.02	0.04	0.04	1
RXRA_HUMAN	15	0.11	0.62	0.35	0.49	0.33	1
SRC_HUMAN	14	0.04	0.27	0.17	0.26	0.22	1
THRB_HUMAN	20	0.03	0.27	0.18	0.24	0.27	1
TRY1_BOVIN	19	0.02	0.39	0.19	0.21	0.23	1
VGFR2_HUMAN	8	0.08	0.23	0.17	0.33	0.33	1

MRC-score (0.33) and rank (0.34) improved with respect to SRC (0.25) when using BEDROC (see Figure 2). Of 36 targets, MRC-score and MRC-rank outperformed SRC runs 33 and 32 times, respectively. The one-tailed t-student's showed that the significance of these results was p<10−6.

MRC-rank outperformed MRC-score 23 times. Despite this trend, which was also observed with other figures of merit, the statistical significance of such a difference was not strong enough to support the exclusive use of MRC-rank.

As it can be seen in Figure 1A, the MRC procedure tended to enhance the extreme behaviors, leading to the depletion of the intermediate region of affinity prediction. In fact, both significant peaks in the MRC distributions, namely the one corresponding to the best predicted binders and the one corresponding to the worst ones, were larger than in the SRC derived distribution. Our “early recognition” oriented approach benefits of the increment of the first peak, leading to a better performance, and ignoring what occurs in the other regions of the distribution.

A comparison between the MRC protocols and the so-called consensus scoring is called for here. Consensus scoring is an accepted approach which was reported to decrease the number of false positives and to improve the hit rate [55], [56]. It combines multiple scoring schemes and it enriches those compounds that are consistently placed in the first positions in each of them. Both MRC and consensus scoring methods can improve the VLS performance with respect to traditional SRC protocol. However, although theoretically possible [57], retrospective studies have clearly demonstrated that consensus scoring was not able to outperform the best single scoring function [58]. Along the same lines, MRC-VLS might be expected to display the same behavior, achieving an overall accuracy that is between the average SRC-VLS performance and the best one. However, in several instances, MRC outperformed the best SRC run. Since the MRC paradigm is based on the coexistence of different conformers, which are mutually excluded in SRC runs, each binder can “select” the most suitable conformation (according to the induced fit paradigm), gaining an exceptionally good score. In this way, MRC achieved higher levels of accuracy than SRC runs and consensus scoring.

### IV. Chemical Diversity in the Topmost Ranking Compound Fraction

**Figure 4:**
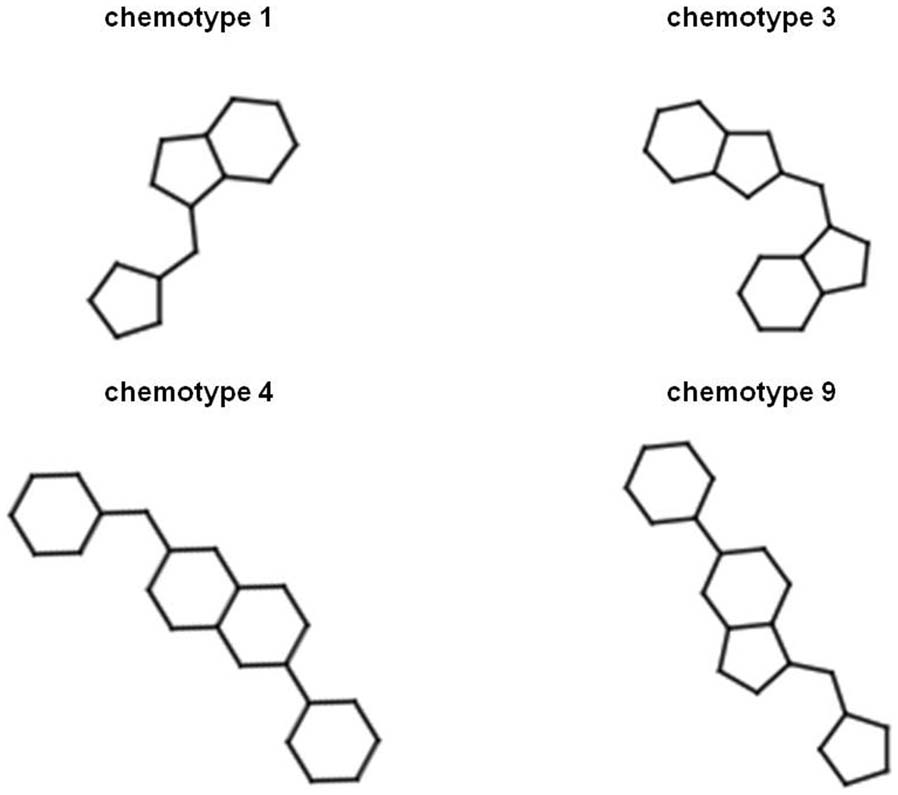
Four different chemotypes enriched in the topmost fraction in single conformer and MRC-VLS studies on basic fibroblast growth factor receptor 1.

The MRC results were also analyzed in terms of chemotypes. A VLS run should be able to enrich as many active compounds as possible, preserving high chemical diversity [54]. In the DUD version used herein, known binders underwent chemical-based cluster analysis and were annotated accordingly. Each binder was converted into a reduced graph and those sharing the same representation were assigned to the same cluster. This specific partitioning scheme was driven by chemical scaffolds and was very robust with respect to local variants and decorations [59]. In the following, we report the case study of FGFR1_HUMAN (i.e. the basic fibroblast growth factor receptor 1) to fully illustrate this concept. For this target, there were 4 receptor conformations, 71 known binders, and 3462 non-binders. The known binders were grouped in 12 chemotypes. Figure 4 reports the chemotypes 1, 3, 4, and 9, which are those relevant to this case study. Table 5 shows that each FGFR1_HUMAN conformer enriched between 2 and 5 binders in the top 36 positions (top 1%). In three cases (PDBid 1AGW, 1FGI, and 1FGK), these binders were representative of chemotypes 1 and 3, and in one case (PDBid: 2FGI) of chemotypes 4 and 9. MRC-score placed 2 binders representative of chemotypes 3 and 9 in the top 1%. Finally, MRC-rank placed 7 binders representative of chemotypes 1, 3, 4, and 9 among the 36 best ranked molecules. The improvement in terms of enrichment with respect to SCR-VLS was absent in MRC-score and modest in MRC-rank. However, when the same results were analyzed in terms of diversity, MRC-score performed at the same level of the best SRC-VLS, while MRC-rank doubled the number of SRC-VLS retrieved scaffolds.

**Table 5:** Chemotype enrichment in different basic fibroblast growth factor receptor 1 conformers.

PDBid	N. of Bindersin theTopmost 1%	N. of Chemotypesin theTopmost 1%	Chemotypesin theTopmost 1%
1AGW	4	2	1–3
1FGI	2	2	1–3
1FGK	5	2	1–3
2FGI	2	2	4–9
MRC-score	2	2	3–9
**MRC-rank**	**7**	**4**	**1–3–4–9**

**Figure 5:**
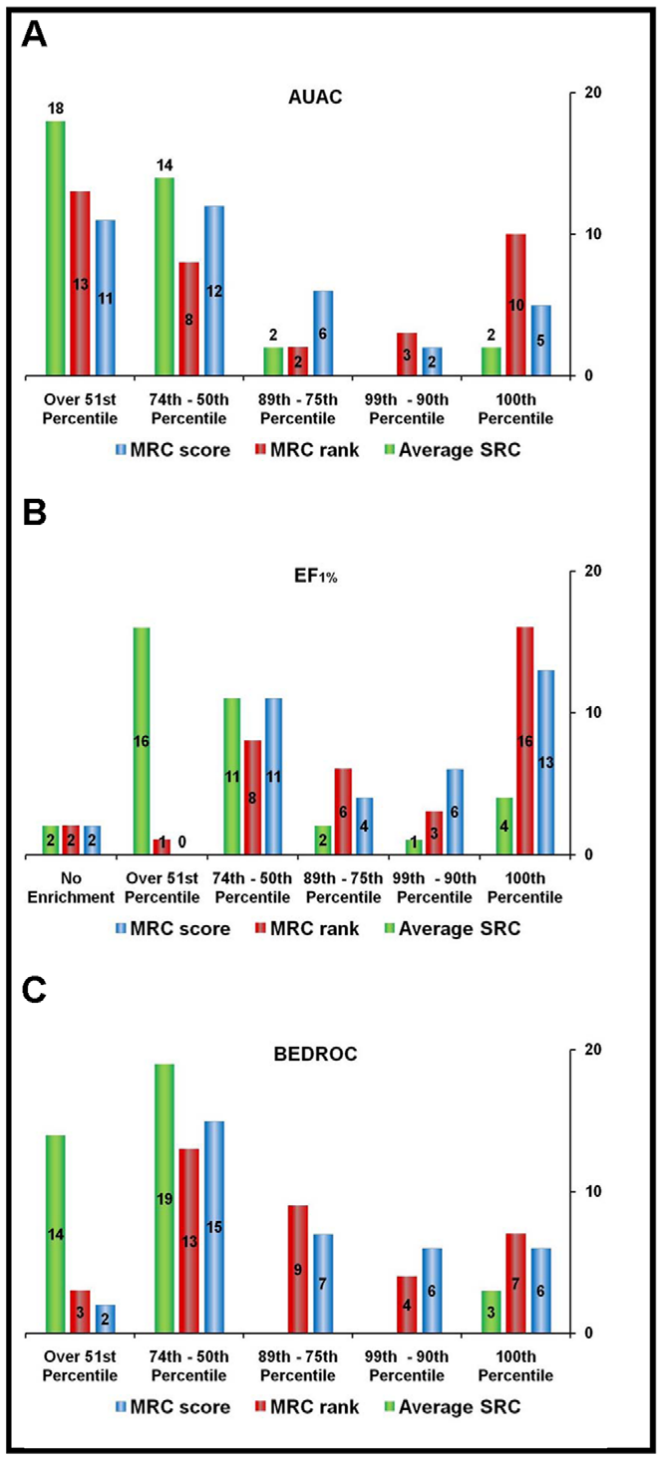
Distribution of the results of MRC-VLS runs obtained by considering only one representative binder for each chemotype in the ligand set. Results are reported according to different figures of merit. Blue histograms represent MRC-score results, red histograms represent MRC-rank results. The average SRC performance is reported (green histograms) as a term of comparison. A) Histograms representing the distribution of the results according to AUAC. B) Histograms representing the distribution of the results according to EF_1%_. C) Histograms representing the distribution of the results according to BEDROC.

To investigate the ability of MRC-VLS protocols to preserve diversity, the results were also analyzed considering only the contribution of the best-ranked binder of each cluster to the enrichment [54]. The other members of the same cluster were labeled as non-binders and their contributions neglected. This strategy greatly reduced the noise due to overrepresented scaffolds. Figure 5 reports the complete distribution of the results considering chemotypes only. When AUAC was used as a figure of merit (Figure 5A), MRC-rank outperformed or performed as well as any single conformer for 10 targets, while MRC-score was in the 100^th^ percentile 5 times. Altogether, MRC-rank and MRC-score were above the 50^th^ percentile in 23 and 25 instances, respectively. For HMDH_HUMAN and ESR1_AG_HUMAN, due to the exceptionally high performance of SRC-VLS, the MRC results were in the 1^st^ percentile even though calculated areas were above 0.8. From a chemical diversity standpoint, the AUAC results suggested that MRC-VLS was still beneficial, even though improvement with respect to single conformers was reduced. However, the scenario was completely different if we analyzed the results in terms of EF_1%_ (Figure 5B). In all but one case (NRAM_INBEE, MRC-rank EF_1%_ below the 50^th^ percentile), MRC-VLS based approaches were above the 50^th^ percentile. In particular, MRC-rank was in the 100^th^ percentile 16 times, while MRC-score was in the 100^th^ percentile 13 times. Dissimilar chemotypes were specifically recognized and assigned a good score/rank by different conformers and, when the results were combined together, the final EF_1%_ was synergistically boosted. In fact, different chemotypes reflect chemically different binders that likely require different receptor conformations to be suitably bound into the binding pocket. This is accounted for with our MRC-VLS approach. The BEDROC results reflect the balanced between the moderate improvement in overall separation and the significant enhancement in early recognition that can be obtained using MRC-VLS (see Figure 5C). In fact, MRC-rank provided results that were above the 50^th^ percentile in 33 targets. But it outperformed or matched the results of the best SRC-VLS in only 7 targets. Similarly, MRC-score was placed over the 50^th^ percentile 34 times, but placed in the 100^th^ percentile only 6 times. These results prove that our MRC-VLS approach increased the number of active molecules among the top scorers (Figure 3B) and, more importantly, enhanced the chemical diversity of true binders. The latter represents the major novelty and added value of our approach to hit identification campaigns.

### Are co-crystals artificially inflating the beneficial role of MRC?

**Figure 6:**
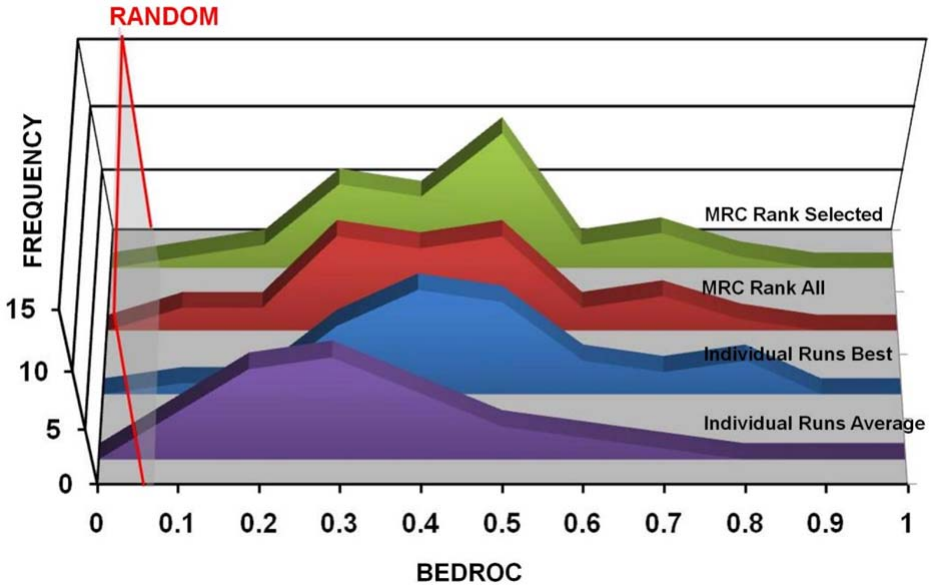
Frequency distributions describing the performance of different protocols, as assessed by the BEDROC metric, with α = 20. The red bordered transparent grey rectangle represents the threshold of randomness. The indigo area represents the frequency distribution of the results for the individual runs (average), the blue area represents the frequency distribution of the results for the best single performing conformer from each ensemble, the red area represents the frequency distribution of the results for the MRC-rank including all available conformer for each ensemble, and the green area represents the frequency distribution of the results for the MRC-rank, dropping the conformers that provide a positive score for each ligand.

Because DUD and the experimental Flexible Pocketome were originally compiled independently, they partially overlap. Some of the receptor conformers used here were actually extracted from co-crystals bound to known binders in the set (see also Table S3 in the Supporting Information). Since crystal structures of holo proteins may retain a strong memory of their cognate ligands [31], self-docking could artificially improve the final results. For this reason, we decided to filter the ligand set by excluding from the annotated binders: i) all the molecules co-crystallized with one of the receptor variants and ii) all the binders belonging to the same chemotype of a co-crystallized molecule (the chemotype of co-crystallized molecules was defined according to the rules reported in reference 34). Since highly populated chemotypes were more likely to include a co-crystallized molecule, the total number of binders and the diversity of the set were affected by the cognate ligand filtering. On average, one third of the binders, but only one fifth of the chemotypes, were excluded from the set. Three targets (HS9A_HUMAN, KITH_HHV11, and MCR_HUMAN) had to be excluded from the test set for the purpose of this analysis since all of their binders were filtered out. In Figure 6, a complete comparison of the BEDROC values is reported. A very small (and somewhat expected) deterioration of the overall performance could be observed. But, on average, the best single conformer, MRC-score, and MRC-rank VLS provided for the filtered ligand set are very similar to those of the non-filtered counterpart. The average fluctuation was 0.03 for the best single conformers and 0.05 for the MRC approaches. Accordingly, the overall distribution of the results was very similar to the percentile analysis reported in Figure 3 (see also Figure S2 in the Supporting Information). The results quality dropped significantly for GCR_HUMAN and HMDH_HUMAN only, implying that, in these two targets, only co-crystals and closely related molecules could be efficiently separated from non-binders. Interestingly, in several cases, it was possible to detect a performance improvement after co-crystallized ligands and their analogs were eliminated. This depended on the noise generated by highly represented chemotypes, which only provided a satisfactory performance in a limited number of conformers, if at all. For example, the acetylcholinesterase (ACES_TORCA) set of binders encompassed the well-known inhibitor donepezil and twenty variants of the same chemotype. While members of this cluster could be separated quite well from non-binders in receptor conformers displaying the right arrangement of the binding cavity, these 20 molecules were dumped at the rank bottom in all the other pocket variants. Losing the huge amount of noise generated by donepezil and its analogues more than compensated for their positive contributions to the final ranking. If we consider Figure 1A, a small reduction of the leftmost peak was compensated for by a considerable reduction of the peak on the right. This, in turn, translated into a better BEDROC score.

These findings are particularly relevant since they imply that MRC-based approaches can statistically improve the quality of the results in an actual VLS campaign, even in the absence of structures specifically adapted to the molecules under examination. These results are in line with previously reported evidence corroborating the idea that even a limited number of randomly selected variants can outperform the traditional single rigid receptor approach in cross docking and VLS [26]–[28], [33]. In this light, it appears safe to assume that crystal structures are not the only valuable source of receptor conformations that can be used to boost early recognition and chemical diversity in MRC-VLS. The insights gained in this study can be extended to computer-generated variants such as snapshots from molecular dynamics [60] and, as was recently reported, homology models [61], [62].

### Attempts to Select an Optimal Subset of Receptor Conformers

It has already been reported that the beneficial role of MRC in VLS can be improved even further if an optimal subset of receptor structures is selected from among the available conformers [33], [63], [64]. However, it is quite difficult to define such a subset in advance. In our retrospective study, we noticed a perfect correspondence between the runs that yielded positive ICM docking scores for all the molecules in the dataset and those that presented an EF_1%_<1, i.e. those performing worse than a random selection. We were led by this observation to believe that these runs did not contribute any useful information, and we therefore performed the tests again after excluding them. The first consequence was to eliminate three targets from the study, namely ADA_BOVIN, COMT_RAT and PGH2_MOUSE. As a matter of fact, the available conformations of the first two targets were characterized by “bad runs” only, i.e. the yielding of positive scores only. According to our hypothesis, the relative ranking provided by the docking runs should have been of very low significance. Indeed, that was the case. In ADA_BOVIN, each individual run performed much worse than the random selection, while the discriminating ability in COMT_RAT was fairly similar to that of the random selection. In these cases, consistent with the “garbage in, garbage out” byword, the MRC procedure could not distil any useful information from the individual runs. Only two conformations were available for the PGH2 MOUSE target and, of these, one had to be filtered out. In this case, there was no sense applying the MRC method. However, thanks to the filtering protocol, the most capable conformation could be identified (BEDROC = 0.28 versus 0.06 of the discarded one).

If we now consider the remaining targets, MRC-score and MRC-rank with pre-filtering outperformed the corresponding versions without the filtering protocol with a statistical significance (assessed via the one-tailed t-test for paired samples) p<0.07 for every figure of merit considered in this work. The BEDROC measure, which takes into account both early recognition and overall performance, particularly benefited from this protocol because the corresponding statistical significance was p<0.005 and p<0.0001 for MRC-score and MRC-rank, respectively. Even more impressively, in slightly more than 40% of targets, this protocol allowed MRC-rank to perform better than the best of the individual runs. The results for MRC-rank performance after filtering out receptor structures that yield only positive ICM docking scores are reported in Figure 6.

In summary, filtering out those individual runs that provided only positive ICM docking scores seems to be an easy way of removing noise from the calculation and best exploiting the MRC procedure.

### Conclusions

In this work, the role of MRC in VLS has been systematically analyzed on a diverse and challenging test set, and several protocols for exploiting MRC availability are suggested. Before testing our protocols, we first established a baseline to assess the performance of the docking engine. We found it to be appreciable and in line with the literature. This preliminary step was necessary to understand whether or not our protocol provided a real improvement. The protocols we suggested, namely the MRC-score and the MRC-rank, statistically outperform the average single conformation run, and this is particularly true as far as the topmost ranking fraction is concerned. This latter is the most relevant fraction when VLS is considered not just as a standalone exercise, but as part of a drug discovery project. It is not entirely clear whether MRC-rank should be preferred to MRC-score, even if the results reported here seem to point in that direction. From a chemical diversity perspective, we proved that MRC improved not only the number of active molecules enriched in the top fraction, but also the variety of scaffolds. Again, this is crucial for real life drug discovery. Furthermore, we proved that the quality of the results does not depend on a bias introduced by co-crystals. Even with co-crystals excluded from the analysis, MRC still outperforms SRC-VLS. On the other hand, it is reasonable to assume that even more structurally diverse ensembles would increase the likelihood of discovering truly novel scaffolds.

We observed that conformations that yield only positive scores in the docking phase can safely be excluded, leading to a significant improvement in the final results. This is a simple yet practical criterion for making a preliminary selection of the conformers whenever a set of known binders and decoys is available. Each of the figures of merit considered in this study has its own peculiarities and privileged domains of application. In a real VLS scenario, where “early recognition” is often crucial, enrichment factors and BEDROC seem to be the most appropriate to evaluate performance.

Finally, we note that MRC strategies significantly increase the computational burden, since the calculation time scales linearly with the number of conformers. However, docking engines purposely developed to integrate MRC in standard protocols were recently reported and will help limit the impact of this issue [32], [65], [66].

## Supporting Information

Figure S1 Structural comparison with cognate ligands A) Inhibitor **1** at the binding site of SRC kinase (PDBid 1YOL). Inhibitor **1** and the binding site residues are reported explicitly in ball and stick representation. Inhibitor **1** alpha carbons are colored green. As a term of comparison, the cognate ligand CGP77675 is reported explicitly in ball and stick representation with dull grey carbon atoms. The boundaries of the binding site are highlighted by a semi-transparent white mesh. Intermolecular hydrogen bonds are reported with dotted lines. B) Modulator **2** at the binding site of Progesterone receptor (PDBid:2OVH). Modulator **2** and the binding site residues are reported explicitly in ball and stick representation. Modulator **2** alpha carbons are colored green. As a term of comparison, the cognate ligand Asoprisnil is reported explicitly in ball and stick representation with dull grey carbon atoms. The boundaries of the binding site are highlighted by a semi-transparent white mesh. C) Tolrestat (**3**) at the binding site of aldose reductase (PDBid: 2FZB). Tolrestat and the binding site residues are reported explicitly in ball and stick representation. Tolrestat alpha carbons are colored green. As a term of comparison, the cognate ligand IDD552 is reported explicitly in ball and stick representation with dull grey carbon atoms. The boundaries of the binding site are highlighted by a semi-transparent white mesh.
(PDF)

Figure S2 Performance comparison of different protocols, as assessed by the BEDROC metric, with α = 20, including and excluding co-crystallized ligands. For each target, six histograms are reported: best single conformer, all ligands – red; best single conformer, no co-crystals – yellow; MRC score, all ligands – blue; MRC score, no co-crystals – white; MRC rank, all ligands – green; MRC score, no co-crystals – orange.
(PDF)

Table S1 List of PDB structures included in the test set.
(PDF)

Table S2 Distribution of the results expressed by EF_10%_.
(PDF)

Table S3 Known binders and co-crystallized ligands overlap.
(PDF)
