# Supplementary figures and images for: Systematic Exploitation of Multiple Receptor Conformations for Virtual Ligand Screening
Source: PLoS One. 2011 May 17;6(5):e18845. doi: 10.1371/journal.pone.0018845 (PMC3098722; doi:10.1371/journal.pone.0018845)

**Figure S1**

**A**

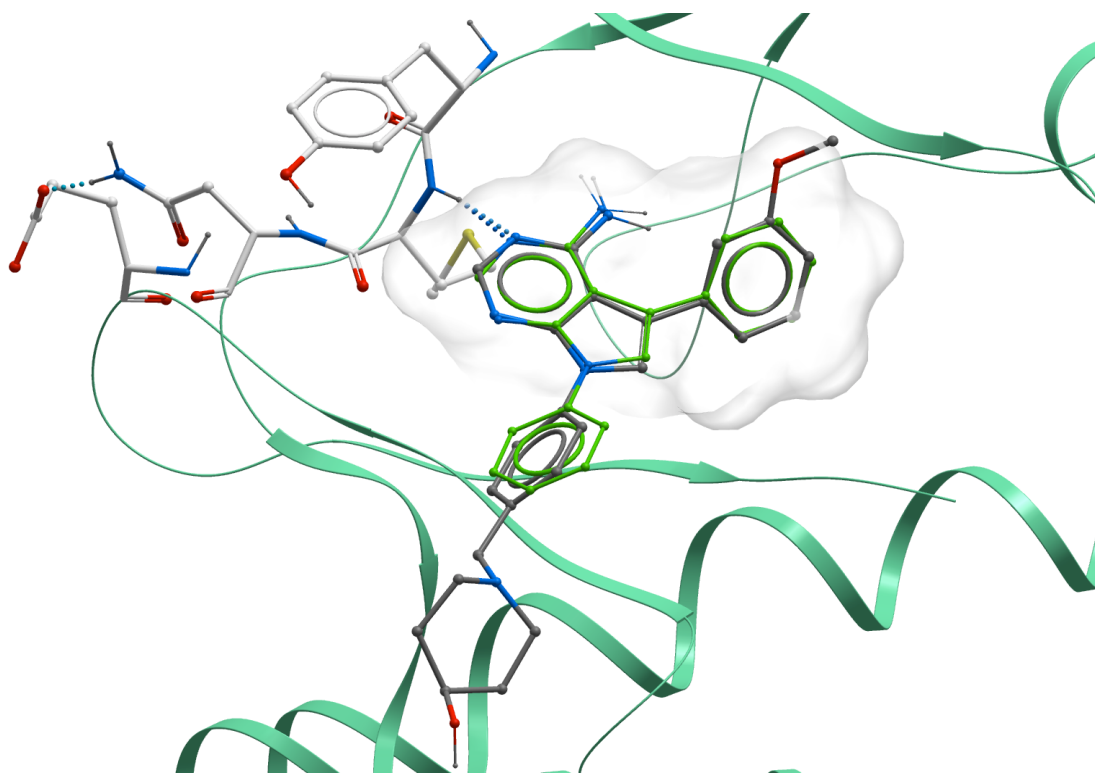

**B**

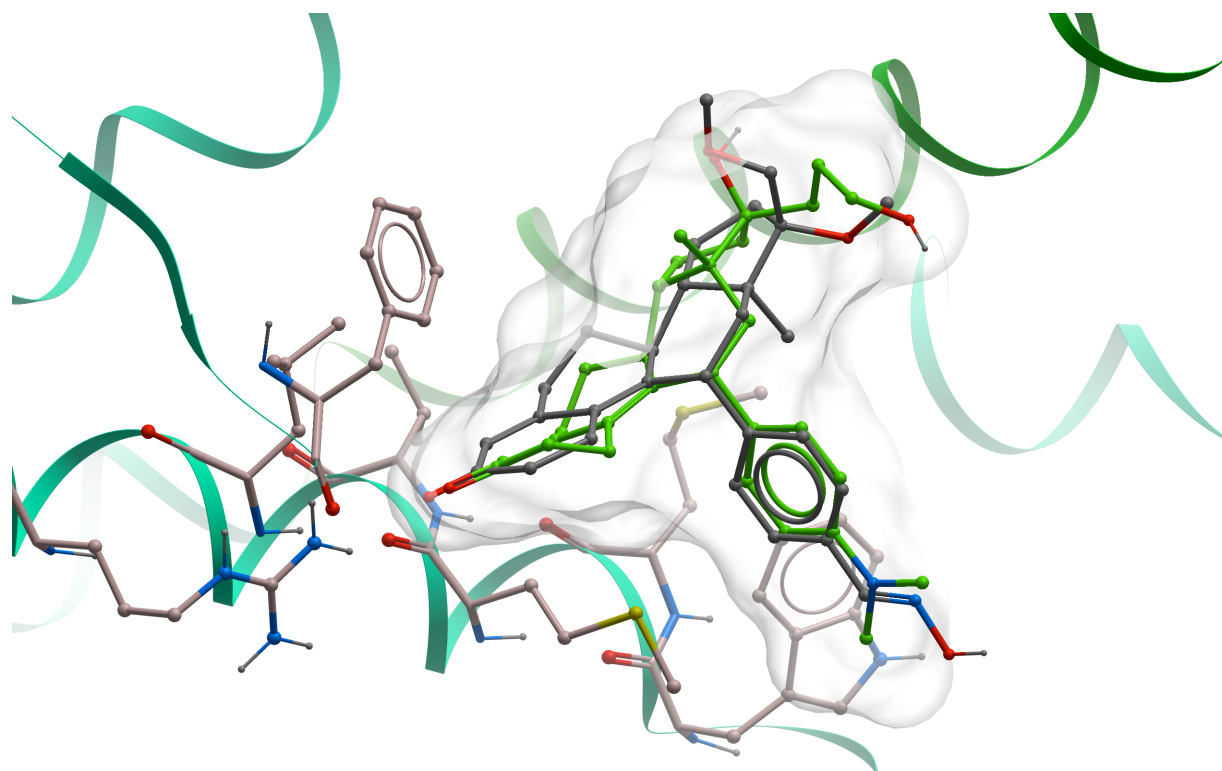

**C**

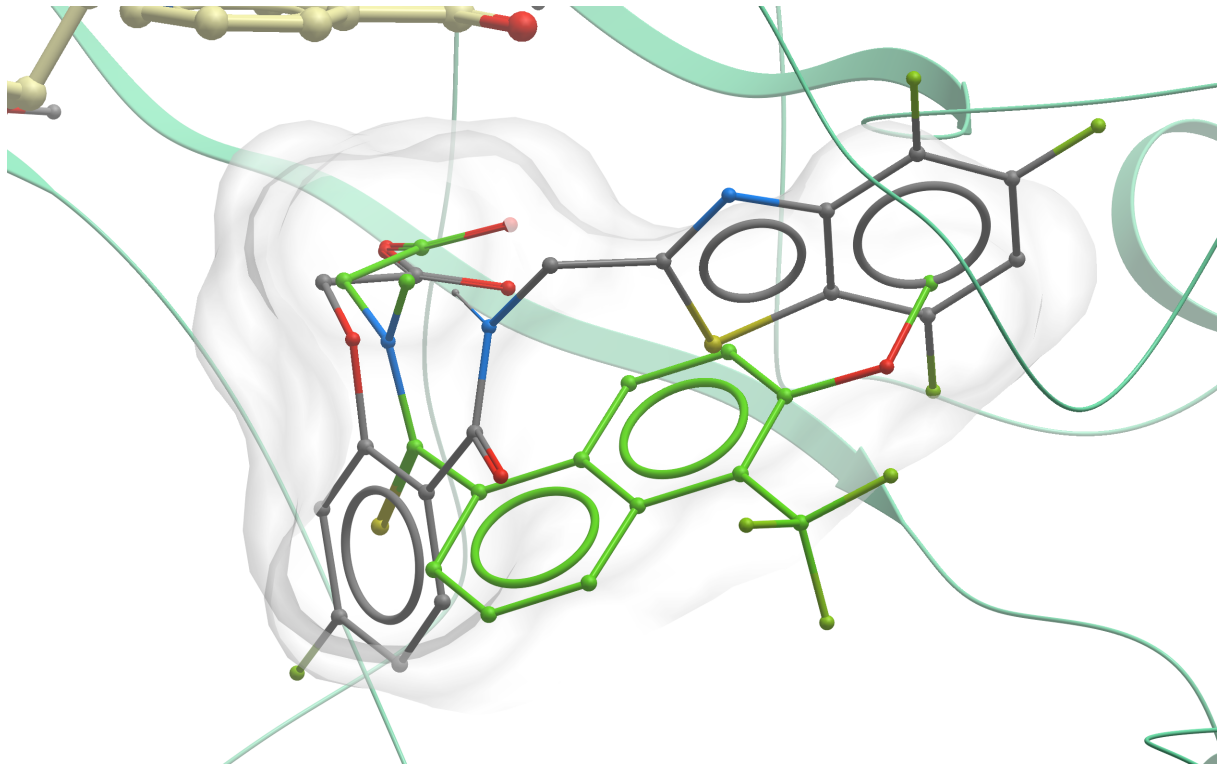

Supplement: Figure S1 — Structural comparison with cognate ligands A) Inhibitor 1 at the binding site of SRC kinase (PDBid 1YOL). Inhibitor 1 and the binding site residues are reported explicitly in ball and stick representation. Inhibitor 1 alpha carbons are colored green. As a term of comparison, the cognate ligand CGP77675 is reported explicitly in ball and stick representation with dull grey carbon atoms. The boundaries of the binding site are highlighted by a semi-transparent white mesh. Intermolecular hydrogen bonds are reported with dotted lines. B) Modulator 2 at the binding site of Progesterone receptor (PDBid:2OVH). Modulator 2 and the binding site residues are reported explicitly in ball and stick representation. Modulator 2 alpha carbons are colored green. As a term of comparison, the cognate ligand Asoprisnil is reported explicitly in ball and stick representation with dull grey carbon atoms. The boundaries of the binding site are highlighted by a semi-transparent white mesh. C) Tolrestat (3) at the binding site of aldose reductase (PDBid: 2FZB). Tolrestat and the binding site residues are reported explicitly in ball and stick representation. Tolrestat alpha carbons are colored green. As a term of comparison, the cognate ligand IDD552 is reported explicitly in ball and stick representation with dull grey carbon atoms. The boundaries of the binding site are highlighted by a semi-transparent white mesh. (PDF) [file pone.0018845.s001.pdf]

Figure S2

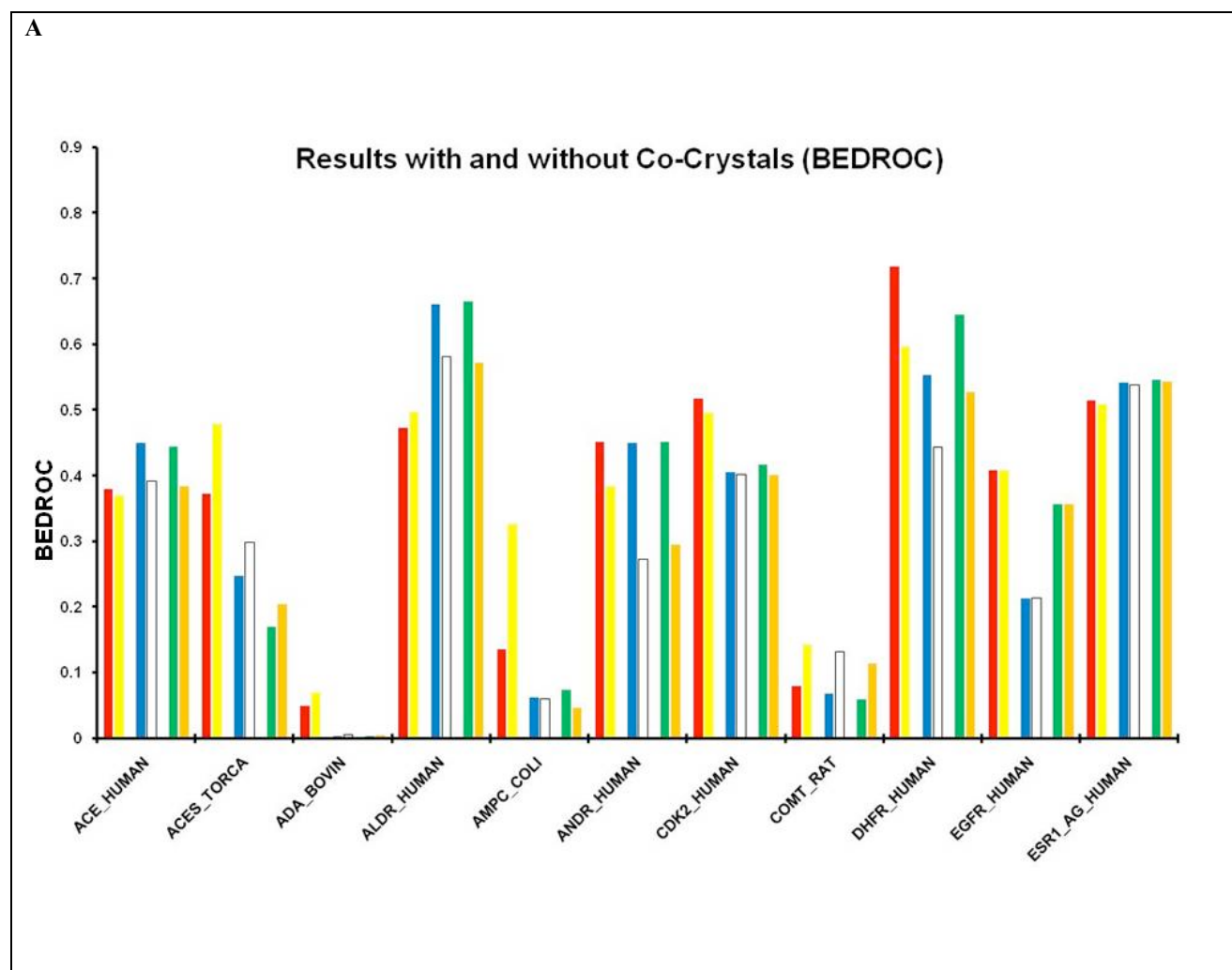

**B**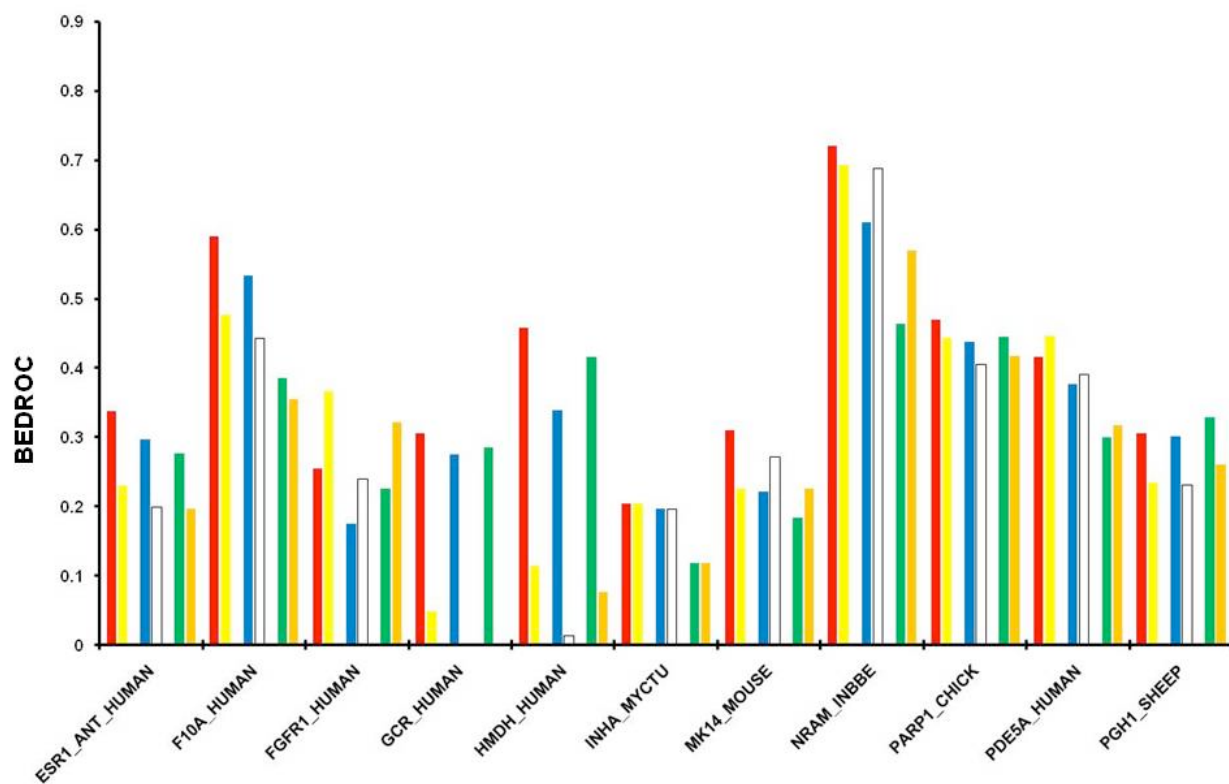

C

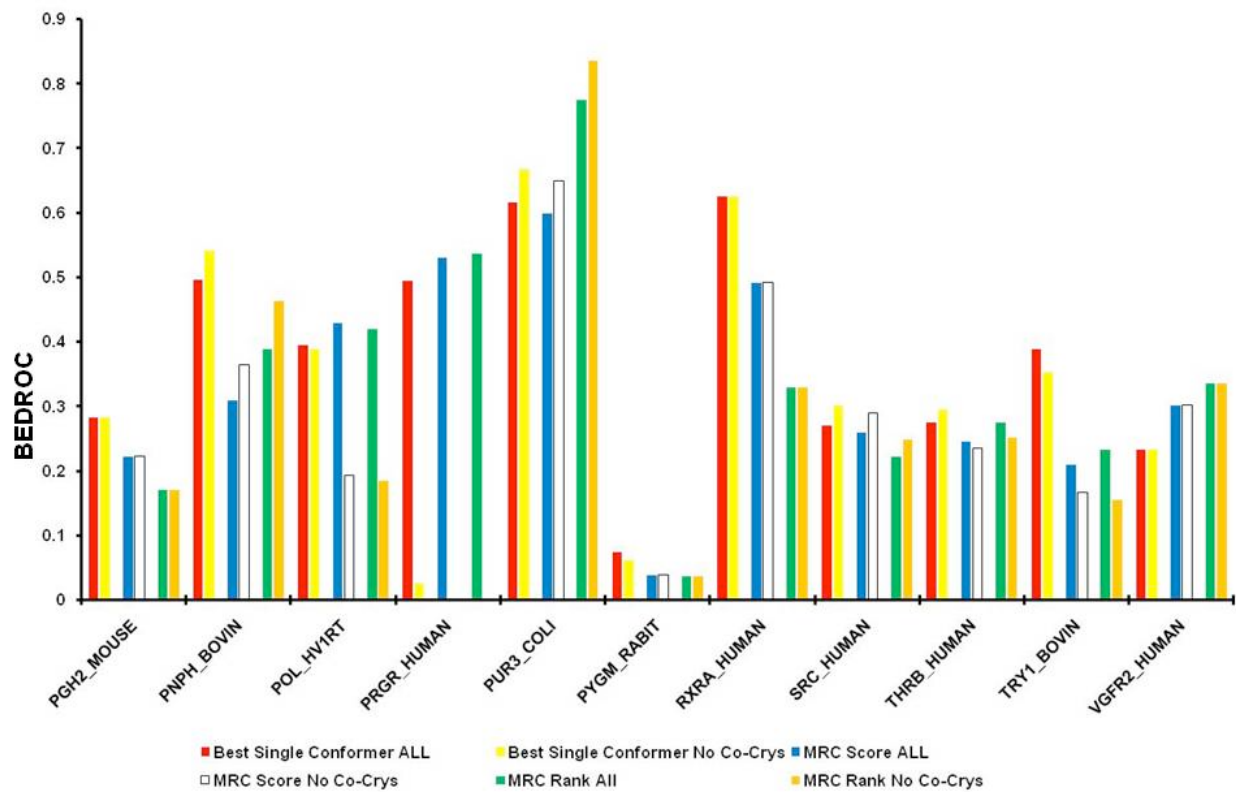

Supplement: Figure S2 — Performance comparison of different protocols, as assessed by the BEDROC metric, with α = 20, including and excluding co-crystallized ligands. For each target, six histograms are reported: best single conformer, all ligands – red; best single conformer, no co-crystals – yellow; MRC score, all ligands – blue; MRC score, no co-crystals – white; MRC rank, all ligands – green; MRC score, no co-crystals – orange. (PDF) [file pone.0018845.s002.pdf]
