# Supplementary material for: Systematic Exploitation of Multiple Receptor Conformations for Virtual Ligand Screening
Source: PLoS One. 2011 May 17;6(5):e18845. doi: 10.1371/journal.pone.0018845 (PMC3098722; doi:10.1371/journal.pone.0018845)
Supplement: Table S1 — List of PDB structures included in the test set. (PDF) [file pone.0018845.s003.pdf]

Table S1

TARGET	PDBids
ACE_HUMAN	1O86, 1O8A, 1UZE, 1UZF, 2OC2, 3BKK, 3BKL
ACES_TORCA	1EVE, 1H22, 1UT6, 1E66, 1ODC, 1ZGB, 1ZGC, 1DX6, 1GPN, 1W4L, 1GPK, 1U65, 1GQR, 2ACK, 1CFJ, 1QTI, 1VOT, 1EA5, 1W6R, 1H23
ADA_BOVIN	1WXY, 1NDV, 1V7A, 1NDZ, 1V79, 1NDW, 1KRM, 1NDY, 1O5R, 1QXL, 1UML, 1VFL, 2E1W
ALDR_HUMAN	1PWL, 1T40, 1EL3, 2NVC, 1IEI, 2IKJ, 2FZB, 2IKI, 2NVD, 1Z89, 1PWM, 1X97, 1X98, 2AGT, 2IKG
AMPC_COLI	1XGJ, 1XGI, 1L2S, 2HDS, 2HDU, 1C3B, 1FCO, 1FSW, 1FSY, 1GA9, 1IEL, 1IEM, 1KDS, 1KDW, 1KE0, 1KE3
ANDR_HUMAN	2AM9, 1E3G, 1T5Z, 1T63, 1T65, 1XOW, 1XQ3, 2AMA, 2AMB, 2AO6, 2AX9, 2AXA, 2PIO, 2PIP, 2PIQ, 2PIR, 2PIT, 2PIU, 2PIV, 2PIX, 2PKL, 2PNU, 2Q7I, 2Q7J, 3B5R, 3B65, 3B66, 3B67, 3B68
CDK2_HUMAN	1YKR, 2B54, 1H1S, 2IW6, 2UZL, 2G9X, 2UUE, 1H1R, 1OI9, 2BPM, 1E9H, 1H1Q, 2C5P, 1VYW, 2UZE, 2EXM, 1P5E, 2C5N, 2C5O, 2C5O, 1FIN, 1H1P, 1H26, 1H27, 1OIY, 1OKV, 1OKW, 1PKD, 1QMZ, 1W98, 2CCH
COMT_RAT	1H1D, 2CL5, 1VID
DHFR_HUMAN	1KMS, 1KMV, 1OHJ, 1PD8, 1U72, 2C2S
EGFR_HUMAN	1XKK, 2EB2, 2ITN, 2ITV, 2RGP, 3BEL
ESR1_AG_HUMAN	2P15, 1L2I, 1ZKY, 2FAI

ESR1_ANT_HUMAN	2OUZ, 3ERT, 1UOM, 1SJ0, 1XP9, 1XPC, 1YIM, 1ERR, 1XP1, 1XP6, 1XQC, 2JFA, 1YIN, 2AYR
F10A_HUMAN	1NFU, 1NFY, 2J2U, 2J34, 2J38, 1F0R, 1F0S, 1IQM, 1XKA, 2CJI, 2D1J, 2J94, 2J95, 2UWO, 1LPK, 1LPZ, 1NFW, 2BOK, 2UWL, 2UWP
FGFR1_HUMAN	1AGW, 1FGI, 1FGK, 2FGI
GCR_HUMAN	1M2Z, 1NHZ, 3E7C, 3BQD
HMDH_HUMAN	1HWI, 1HWJ, 1HWL, 1HWK, 1HW8, 1HW9, 2Q1L, 2Q6B, 2Q6C
HS9A_HUMAN	1UYF, 1UYE, 2H55, 1UYD, 2BYI, 2FWY, 2FWZ, 1UY6, 1UYI, 1UYM, 1YC1, 2BSM, 2UWD, 1UYH, 2BT0, 2BYH, 2CDD, 1UYC, 1UYK, 1UY9
INHA_MYCTU	2H7M, 2H7N, 2H7P, 2B37, 2H7L, 2H7I, 1ENY, 1P45, 2AQ8, 2AQH, 2AQI, 2AQK, 2B35, 2NSD
KITH_HHV11	1KI4, 1E2N, 1E2K, 1OF1, 1E2P, 1E2M, 1E2H, 1E2L, 1E2I, 1E2J, 1KI2, 1KI3, 1KI6, 1KI7, 1KI8, 1KIM, 1P7C, 1QHI, 2KI5
MCR_HUMAN	2AA2, 2AAX, 2AB2, 1YA3, 1Y9R, 2AA7, 2AA5, 2A3I, 2AA6, 2ABI, 2OAX
MK14_MOUSE	1W83, 1YW2, 2GTN, 1WBN, 2BAL, 2GHL, 2GTM, 1A9U, 1M7Q, 1WBS, 1WBT, 1ZZ2, 2BAJ, 2EWA, 2GFS, 2GHM, 1W82, 1BL7, 1WBV
NRAM_INBBE	1B9V, 1B9T, 1B9S, 1INF, 1IVB, 1A4G, 1A4Q, 1INV, 1NSB, 1NSC, 1NSD
PARP1_CHICK	1EFY, 2PAX, 1PAX, 1A26, 2PAW, 3PAX
PDE5A_HUMAN	

	1XOZ, 2CHM, 1TBF, 1UHO, 1RKP, 1UDT, 1XP0, 2H42, 1T9S, 2H40, 2H44
PGH1_SHEEP	2AYL, 1Q4G
PGH2_MOUSE	3PGH, 1CVU
PNPH_BOVIN	2AI2, 1V48, 1LV8, 1A9P, 1A9O, 1LVU, 1A9Q, 1A9R, 1A9S, 1A9T, 1B8N, 1B8O, 1FXU, 1PBN, 1VFN, 2AI1, 2AI3, 3PNP, 4PNP
POL_HV1RT	1EP4, 1JLA, 2BE2, 1S9E, 1C1B, 1C0U, 1VRU, 1C1C, 1JLG, 1RT1, 1S1T, 1TKT, 1FK9, 1REV, 2OPP, 1RT2, 1JKH
PRGR_HUMAN	1A28, 2OVH, 1SR7, 1SQN, 1ZUC, 2OVM
PUR3_COLI	1C2T, 1C3E, 1CDE
PYGM_RABIT	2IEI, 1C50, 1WUT, 1WV0, 1WV1, 1Z6P, 1C8K, 1C8L, 1E1Y, 1WUY, 2AMV, 2IEG, 1Z62, 1UZU, 1K06, 2GM9, 2GJ4, 1FU4, 1XKX, 1XL1
RXRA_HUMAN	1RDT, 1MVC, 1XVP, 1FM6, 1K74, 1FM9, 1DKF, 1MZN, 1FBY, 1G1U, 1G5Y, 1MV9, 2P1T, 2P1U, 2P1V
SRC_HUMAN	1YOL, 1YOL, 2BDF, 2BDJ, 1Y57, 2OIQ, 2H8H, 1FMK, 1YI6, 1YOJ, 2HWO, 2HWP, 2PTK, 2SRC
THRB_HUMAN	1KTT, 1K22, 1MU6, 2BXT, 2JH0, 1BHX, 1D4P, 1G32, 1MU8, 1MUE, 1O5G, 1QBV, 1T4U, 1TOM, 1ZGI, 2A2X, 2FEQ, 2FES, 2JH5, 2JH6
TRY1_BOVIN	1F0T, 1V2O, 1Y59, 1Y5A, 1Y5B, 1Y5U, 1K1P, 1G36, 1O2R, 1O2U, 1QBN, 1V2N, 1K1L, 1MTW, 1O2T, 1AZ8, 1BJV, 1F0U, 1GJ6
VGFR2_HUMAN	2P2I, 1Y6A, 2P2H, 1Y6B, 1VR2, 1YWN, 2OH4, 2QU6
