# Supplementary material for: Systematic Exploitation of Multiple Receptor Conformations for Virtual Ligand Screening
Source: PLoS One. 2011 May 17;6(5):e18845. doi: 10.1371/journal.pone.0018845 (PMC3098722; doi:10.1371/journal.pone.0018845)
Supplement: Table S2 — Distribution of the results expressed by EF10%. (PDF) [file pone.0018845.s004.pdf]

Table S2

TARGET	Number of Conformers	Min $EF_{10\%}$	Max $EF_{10\%}$	Mean $EF_{10\%}$	MRC Score $EF_{10\%}$	MRC Rank $EF_{10\%}$	Ideal $EF_{10\%}$
ACE_HUMAN	7	3.0	4.0	3.5	5.0	5.0	
ACES_TORCA	21	0	5.0	1.5	3.7	2.6	
ADA_BOVIN	13	0	0.9	0.1	0	0	
ALDR_HUMAN	15	1.1	5.0	3.2	7.3	7.3	
AMPC_COLI	16	0	4.8	0.6	0	4.8	
ANDR_HUMAN	29	1.2	5.6	3.9	5.1	5.9	
CDK2_HUMAN	30	1.3	6.6	3.4	5.7	5.7	
COMT_RAT	3	0	0.9	0.6	1.8	0.9	
DHFR_HUMAN	6	4.5	7.9	5.6	7.6	8.3	
EGFR_HUMAN	6	1.2	4.8	2.9	2.6	4.7	
ESR1_AG_HUMAN	4	4.7	6.5	5.8	6.5	6.5	
ESR1_ANT_HUMAN	13	0	3.9	2.5	3.3	3.3	
F10A_HUMAN	20	0.8	6.2	3.2	5.9	5.3	
FGFR1_HUMAN	4	1.1	4.1	1.9	2.4	3.4	
GCR_HUMAN	4	0	3.1	1.3	2.8	2.8	
HMDH_HUMAN	9	2.0	4.8	3.5	4.0	4.4	
HS9A_HUMAN	20	0	3.9	1.2	1.7	2.2	
INHA_MYCTU	14	0	3.5	1.6	2.8	1.7	
KITH_HHV11	19	0	4.5	2.4	3.2	2.7	
MCR_HUMAN	11	6.1	7.7	7.1	6.9	6.9	
MK14_MOUSE	19	0	4.2	1.4	2.6	2.6	
NRAM_INBBE	11	5.3	8.2	7.0	7.7	6.5	
PARP1_CHICK	6	4.8	7.1	5.9	7.1	6.8	
PDE5A_HUMAN	11	3.1	6.1	4.7	5.4	4.2	
PGH1_SHEEP	2	3.0	3.9	3.5	3.9	3.9	
PGH2_MOUSE	2	0.9	4.5	2.7	3.5	3.2	
PNPH_BOVIN	19	0.8	6.0	2.9	5.2	5.2	
POL_HV1RT	18	1.2	4.4	3.2	4.1	6.2	
PRGR_HUMAN	6	3.2	5.4	4.2	5.4	5.9	
PUR3_COLI	3	5	7.5	5.8	6.2	7.5	
PYGM_RABIT	20	0	1.0	0.3	0.4	0.6	
RXRA_HUMAN	15	1.6	8.3	5.0	6.7	5.5	
SRC_HUMAN	14	0.8	3.6	2.1	3.1	3.2	
THRB_HUMAN	20	0.9	3.9	2.7	3.9	3.9	
TRY1_BOVIN	19	0	6.7	3.7	2.2	2.2	
VGFR2_HUMAN	8	1.2	3.1	2.3	3.1	4.4	
