# Supplementary material for: Systematic Exploitation of Multiple Receptor Conformations for Virtual Ligand Screening
Source: PLoS One. 2011 May 17;6(5):e18845. doi: 10.1371/journal.pone.0018845 (PMC3098722; doi:10.1371/journal.pone.0018845)
Supplement: Table S3 — Known binders and co-crystallized ligands overlap. (PDF) [file pone.0018845.s005.pdf]

Table S3

**TARGET**	**Conformers**	**Binders**	**Binders Chemotypes**	**Non co-crystallized binders**	**Non co-crystallized Chemotypes**
ACE_HUMAN	7	46	18	35	17
ACES_TORCA	21	99	18	63	14
ADA_BOVIN	13	23	8	15	7
ALDR_HUMAN	15	46	14	17	9
AMPC_COLI	16	21	6	5	4
ANDR_HUMAN	29	68	10	33	8
CDK2_HUMAN	30	47	32	37	27
COMT_RAT	3	11	2	5	2
DHFR_HUMAN	6	190	14	49	12
EGFR_HUMAN	6	365	40	365	40
ESR1_AG_HUMAN	4	63	10	62	9
ESR1_ANT_HUMAN	13	18	8	9	4
F10A_HUMAN	20	64	19	30	15
FGFR1_HUMAN	4	71	12	28	10
GCR_HUMAN	4	32	9	23	7
HMDH_HUMAN	9	25	4	15	2
HS9A_HUMAN	20	23	4	0	0
INHA_MYCTU	14	57	23	57	23
KITH_HHV11	19	22	7	0	0
MCR_HUMAN	11	13	2	0	0
MK14_MOUSE	19	137	20	71	18
NRAM_INBBE	11	49	7	7	5
PARP1_CHICK	6	31	7	27	3
PDE5A_HUMAN	11	26	22	24	20
PGH1_SHEEP	2	23	11	20	10
PGH2_MOUSE	2	212	44	211	43
PNPH_BOVIN	19	25	4	20	2
POL_HV1RT	18	34	17	10	8
PRGR_HUMAN	6	22	4	1	1
PUR3_COLI	3	8	5	7	4
PYGM_RABIT	20	52	10	50	9
RXRA_HUMAN	15	18	3	18	3
SRC_HUMAN	14	98	21	86	20
THRB_HUMAN	20	23	14	20	11
TRY1_BOVIN	19	9	7	6	4
VGFR2_HUMAN	8	48	31	48	31
